# Qingchang Wenzhong Decoction Prevents the Occurrence of Intestinal Tumors by Regulating Intestinal Microbiota and Gasdermin E

**DOI:** 10.3389/fphys.2022.917323

**Published:** 2022-07-14

**Authors:** Lingli Ren, Zhengwei Zhang, Wenjing Zhao, Bing Zhao, Xi Chen, Yongxiang Wang, Zhong Chen, Juan Ye, Yang Yang, Peng Cao

**Affiliations:** ^1^ Affiliated Hospital of Integrated Traditional Chinese and Western Medicine, Nanjing University of Chinese Medicine, Nanjing, China; ^2^ Department of Pharmacology, School of Pharmacy, Nanjing University of Chinese Medicine, Nanjing, China; ^3^ Yangtze River Pharmaceutical Group, Taizhou, China; ^4^ Jiangsu Key Laboratory for Pharmacology and Safety Evaluation of Chinese Materia Medica, Nanjing University of Chinese Medicine, Nanjing, China

**Keywords:** intestinal tumor, Qingchang Wenzhong decoction, gut microbiota, gasdermin E, traditional Chinese medicine

## Abstract

**Background:** Intestinal tumors are the third most common malignant tumors worldwide, accounting for approximately 10% of all new cancer cases worldwide. Cancer prevention is a promising way to limit the intestinal tumor incidence rate; however, challenges remain. Qingchang Wenzhong decoction (QCWZD) can clinically treat mild to moderate ulcerative colitis symptoms. Moreover, the mechanism by which it prevents intestinal tumors has not been clarified. In this study, we explored the mechanism by which QCWZD prevents the occurrence of intestinal tumors.

**Methods:** To study the preventive mechanism of QCWZD on intestinal tumors, we used two model mice with azoxymethane/dextran sodium sulfate (AOM/DSS)- and Apc^min/+^-induced intestinal tumor formation. The two models exhibited colitis-associated cancer and familial adenomatous polyposis, respectively. Colon and small intestine tissues were collected and analyzed based on histopathology and immunohistochemistry analyses. Fecal samples were collected, and 16S rRNA sequencing was used to analyze the correlation between intestinal microbiota and the prevention of intestinal tumors.

**Results:** In the AOM/DSS mice, the QCWZD reduced the number and size of tumors, as well as tumor load. Similarly, in the Apc^min/+^ mice, QCWZD can also reduce the number of tumors and the tumor load. The results of 16S rRNA sequencing confirmed that QCWZD altered the composition of intestinal microbiota in mice, a phenomenon that may prevent the occurrence of intestinal tumors by aiding the increase in the abundance of beneficial bacteria, such as *Ralstonia* and *Butyricicoccus*, and reducing that of pathogenic bacteria, such as *Desulfobacterota* and *Bacteroides*, in the intestine. Further, immunohistochemistry reveald that QCWZD can improve the expression of intestinal barrier-related proteins and inhibit pyroptosis-related proteins.

**Conclusions:** QCWZD has the potential to prevent the occurrence of intestinal tumors. The anti-tumor activity may be achieved by regulating the intestinal microbiota, improving the function of the intestinal barrier, and inhibiting GSDME mediated pyroptosis.

## 1 Introduction

Intestinal tumors are major malignant tumors that endanger human health. Worldwide, these have high morbidity and mortality rates (Sung et al., 2021). Colorectal cancer (CRC) has the highest incidence rate among all intestinal tumors. Genetic factors, inflammation, and eating habits are important risk factors for intestinal tumors. Among these, chronic inflammation is closely associated with approximately ¼ of human cancers (Westbrook et al., 2016). In humans, the high incidence rate of intestinal tumors in patients with inflammatory bowel disease suggests that chronic inflammation is closely associated with cancer (Piotrowski et al., 2020). Cancer prevention is an effective way to reverse, inhibit, or prevent the initial stage of carcinogenesis or the progression of precancerous cells to invasive diseases. Currently, preventive drugs used for intestinal tumors mainly include aspirin, statins, and metformin. However, the therapeutic effect of these drugs is uncertain, and their long-term use is associated with the potential risks of systemic adverse reactions and side effects (Rothwell et al., 2018; Katona and Weiss, 2020; Maniewska and Jezewska, 2021). Therefore, the search for drugs that effectively prevent intestinal tumors is still ongoing.

Studies have shown that the intestinal microbiota is closely associated with intestinal tumors (Mima et al., 2017). Intestinal microbiota is involved in various stages of tumor occurrence, development, and metastasis. Therefore, it can be used as a marker for early tumor warning and prognosis prediction and as a potential target for tumor prevention and treatment (Temraz et al., 2019; Ren et al., 2021). Studies have confirmed that several pathogenic bacteria associated with CRC, such as *Escherichia coli* and *Clostridium nucleatum*, promote the occurrence and development of tumors through different mechanisms (Arthur et al., 2012; Rubinstein et al., 2013; Yang et al., 2017). However, some probiotics can prevent and treat intestinal tumors by regulating the immune system, improving intestinal barrier function, and secreting anticancer substances (Ding et al., 2020; Tripathy et al., 2021). In addition, the intestinal microbiota and barrier have a close relationship. Imbalance in the intestinal microbiota will affect the mucous layer of the intestinal barrier system and subsequently affect the mechanical barrier composed of tight junctions (TJs) and adhesive junctions (AJs), intensifying the inflammatory response and occurrence of tumors (Dokladny et al., 2016; Bach Knudsen et al., 2018; Martens et al., 2018). Therefore, the intestinal barrier plays an important role in preventing intestinal tumors.

Pyroptosis is a form of inflammatory cell death, which occurs due to pathogenic infections. Intestinal microbiota can promote the occurrence and development of intestinal tumors by mediating cell death. However, intestinal microbiota can also promote the release of gasdermin E N-terminal (GDSME-N) fragments by activating the inflammatory corpuscles. GDSME-N fragments form pores in the cell membrane, a phenomenon that changes the osmotic pressure of cells, resulting in cell membrane breakdown and pyroptosis. Importantly, lipopolyssacharides in the cytoplasm of Gram-negative bacteria promote the release of GDSME-N fragments, resulting in cytoplasmic swelling and scorch death and by extension, the release of high-mobility group box 1 protein (HMGB1), interleukin (IL)-1β, and IL-18 (Xia et al., 2019; Tan et al., 2021). Hence, bacteria present in tumors can cause cell death and chronic inflammation and promote tumor growth (de Martel et al., 2012; Moskowitz et al., 2021). Gasdermin proteins are the main executors of cell pyroptosis. GSDME cleavage occurs during changes in cell osmotic pressure, leading to cell membrane lysis and pyroptosis (Shi et al., 2015; Wang et al., 2017; Feng et al., 2018). This pore-forming activity leads to cytoplasmic swelling and release of intracellular contents, such as immunogenic damage-associated molecular patterns that include HMGB1 (Boyapati et al., 2016; Rogers et al., 2017; Broz et al., 2020). Previous studies have shown that HMGB1 released by GSDME-mediated pyrolytic epithelial cells can participate in the tumorigenesis of colitis-associated CRC. Therefore, GSDME is closely associated with the occurrence of intestinal tumors.

Qingchang Wenzhong decoction (QCWZD) is a traditional Chinese medicine developed by Li (Mao et al., 2016). It is composed of *Coptis chinensis* Franch, *Curcuma longa* L*.*, *Strobilanthes cusia (Nees)* Kuntze, *Sophora flavescens* Aiton, *Basella alba* L., *Dolomiaea costus*, *Sanguisorba officinalis* L.*,* and *Glycyrrhiza glabra* L. Clinical studies have shown that QCWZD has significant clinical efficacy in patients with mild to moderate ulcerative colitis (UC). In addition, in model rats with dextran sodium sulfate (DSS)-induced UC, QCWZD alleviated inflammation and improved colitis by downregulating the interferon γ-induced protein 10/CXCR3 axis (Mao et al., 2016), upregulating the macrophage-stimulating protein/recepteur d’origine nantais signaling pathway (Mao et al., 2017), promoting the expression of *NLRP12* mediated by the intestinal microbiota, and improving the integrity of the intestinal barrier (Shi et al., 2019; Sun et al., 2019). We used azoxymethane AOM/DSS and Apc^min/+^ mouse models to elucidate the protective mechanism of QCWZD against intestinal tumors. We also studied the role of the intestinal microbiota in intestinal carcinogenesis.

## 2 Materials and Methods

### 2.1 Antibodies and Reagents

DSS (160,110) was purchased from MP Biomedicals, AOM (A5486) was purchased from Sigma-Aldrich. ZO-1 antibody (21773-1-AP), Muc2 antibody (27675-1-AP), ZO-2 antibody (18900-1-AP), IL-18 (10663-1-AP) was purchased from Proteintech. Occludin antibody (BS72035), HMGB1 antibody (BS 1918), NLRP3 antibody (BS9094), IL-1β (BS65005) was purchased from Bioworld. GSDME antibody (A7432) was purchased from ABclonal. E-cadherin antibody (3195S) was purchased from Cell Signaling Technology. Dry ointment powder of QCWZD was provided by the Yangzi River Pharmaceutical Group.

### 2.2 Animals

Male C57BL/6 mice (6–8 weeks old, with a body weight of 20–22g) were purchased from Changzhou Cavins Laboratory Animal Co. Ltd. Apc^min/+^ mice (male, 5 weeks old) were obtained from GemPharmatech Co., Ltd (Nanjing, China). The mice were housed under standard laboratory conditions at the Jiangsu Institute of Traditional Chinese Medicine (room temperature 22 ± 2°C, humidity 50 ± 10%) with a 12 h light/dark cycle. All procedures involving animals were approved by the Institutional Animal Care and Use Committee of the Jiangsu Institute of Traditional Chinese Medicine, and the details of the procedures were drafted in accordance with the guidelines. The experiment was conducted in accordance with the guidelines published by the National Institutes of Health.

**FIGURE 1:**
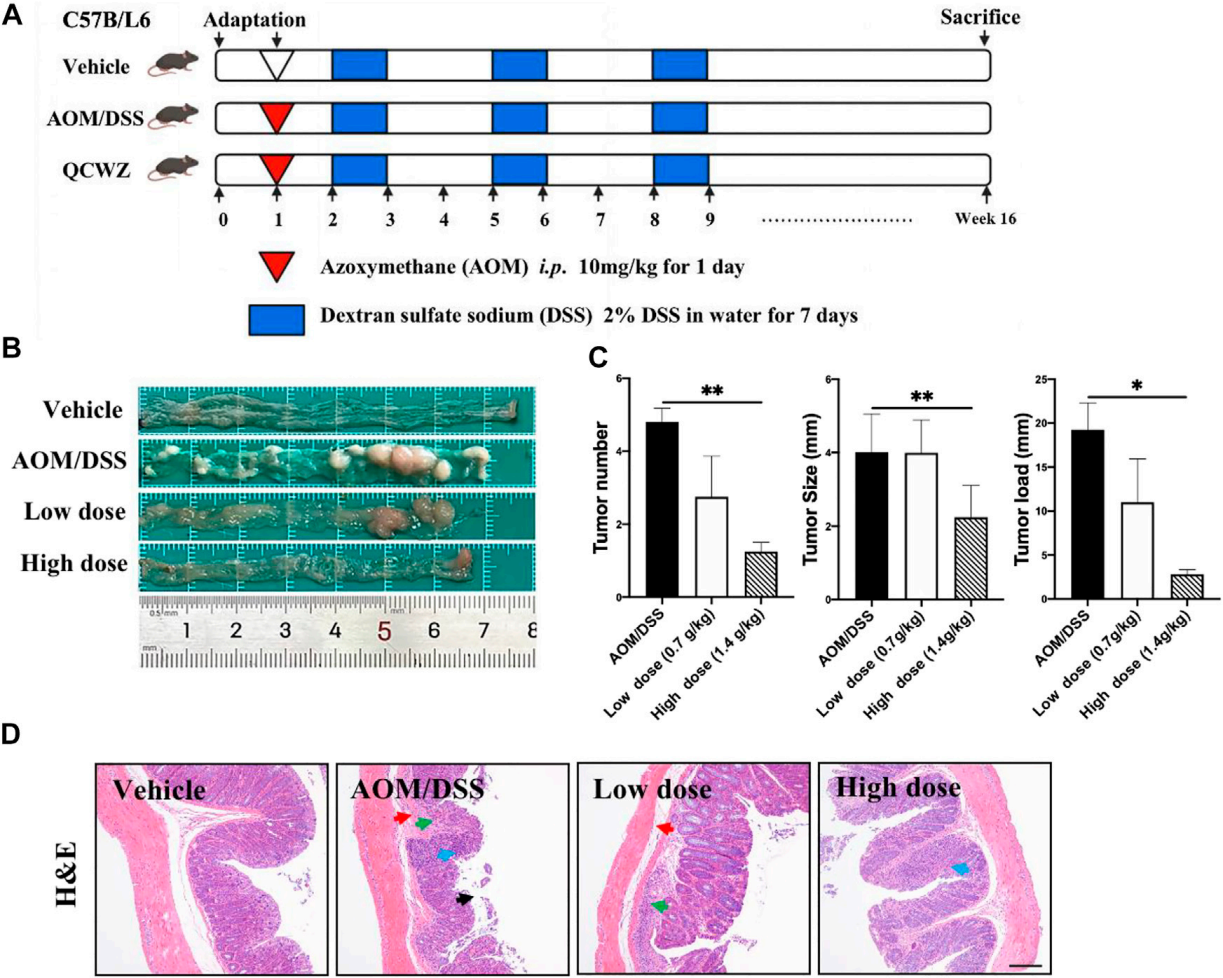
Preventive effect of QCWZD on AOM/DSS-induced colon cancer in C57BL/6 mice. **(A)** AOM/DSS-induced animal model in C57B/L6 mice .**(B)** Colon morphology .**(C)** Measured tumor number, size, and load. **(D)** Representative H&E-stained colorectum sections (magnification: ×200). Black arrow: local necrosis and abcission of intestinal mucosal epithelial cells, blue arrow: focal infiltration of inflammatory cells in the mucosal layer, green arrow: fibrous tissue hyperplasia in the mucosal layer and disappearance of some intestinal glands, red arrow: fibrous connective tissue hyperplasia and increased tissue space in the submucosa; low dose: 0.7 g/kg, high dose: 1.4 g/kg; ^*^ *p* < 0.05, ^**^ *p* < 0.01.

For the mouse model induced by AOM/DSS, the mice were divided into four groups (*n* = 6–8): vehicle group, AOM/DSS group, low-dose QCWZD group (0.7 g/kg) and high-dose QCWZD group (1.4 g/kg). After one week of adaptive feeding, the mice were intraperitoneally injected with 10 mg/kg AOM. Seven days later, the mice were administered 2% DSS dissolved in drinking water for seven consecutive days, followed by 14 days of regular drinking water for recovery. This same cycle was repeated twice, followed by regular drinking water until the 15th week when the mice were killed. The QCWZD treatment group began intragastric treatment on day 0. A schematic diagram of the experimental design is shown in Figure 1A.

For Apc^min/+^ mouse model, mice were divided into three groups (*n* = 6–8): vehicle group, model group and high-dose QCWZD group (1.4 g/kg). The mice were fed a 60% high-fat diet. The QCWZD treatment group was administered QCWZD by gavage from the first day, and the mice were killed at the 14th week. A schematic diagram of the experimental design is shown in Figure 5A.

### 2.3 Haematoxylin and Eosin Staining

The complete intestinal tissue was cut longitudinally, the fecal contents were removed, the tissue was washed with PBS, and fixed with 4% paraformaldehyde overnight. The fixed intestinal tissue was embedded in paraffin blocks into 5 μm-thick slices. Sections were dewaxed and rehydrated using a xylene–ethanol–water gradient system and stained with hematoxylin and eosin (H&E) using a standard protocol. Microscopic observations and pathological examinations were performed.

### 2.4 Immunohistochemistry

The expression levels of MUC2, Occludin, ZO-1, ZO-2, E-cadherin, NLRP3, GSDME, HMGB1, IL-1β, IL-18 in paraffin-embedded sections (5 μM) of intestinal tissues were evaluated using IHC with specific antibodies. After dewaxing, the sections were antigen repaired, background blocked, and then incubated with primary antibody at 4°C overnight. Biotin-labeled secondary antibody and streptavidin horseradish peroxidase (HRP) were incubated at room temperature for 30 min. Immune response was detected with 3-3-diaminobenzidine and counterstained with hematoxylin. The sections were then observed under an optical microscope. Image analysis was performed by blind method and analyzed with iamge Pro plus6 software to score the immune response of samples and calculate the area density of each sample. Area density is the cumulative optical density (IOD)/tissue area of the area to be tested. The cumulative optical density value is the integral of the optical density of all the positive signals. Dividing by the area product of the area to be tested can reflect the positive number and depth, which are in direct proportion.

### 2.5 16S rRNA Gene Sequencing and Data Analysis

Mouse feces were collected, quickly frozen in liquid nitrogen, and stored at − 80°C. Microbial DNA was extracted from the samples using the E. Z.N.A.^®^ Soil DNA Kit (Omega Bio-tek, Norcross, GA, United States) according to the manufacturer’s protocols. The V4-V5 region of the bacterial 16S ribosomal RNA gene was amplified by PCR (95°C for 2 min, followed by 25 cycles at 95°C for 30 s, 55°C for 30 s, and 72°C for 30 s, and a final extension at 72°C for 5 min) using the primers 515F 5′-barcode- GTGCCAGCMGCCGCGG)-3′ and 907R 5′-CCGTCAATTCMTTTRAGTTT-3′, where the barcode is an eight-base sequence unique to each sample. PCR reactions were performed in triplicate in a 20 μl mixture containing 4 μl of 5 × FastPfu Buffer, 2 μl of 2.5 mM dNTPs, 0.8 μl of each primer (5 μM), 0.4 μl of FastPfu Polymerase, and 10 ng of template DNA. Amplicons were extracted from 2% agarose gels and purified using an AxyPrep DNA Gel Extraction Kit (Axygen Biosciences, Union City, CA, United States) according to the manufacturer’s instructions. Purified PCR products were quantified by Qubit^®^3.0 (Life Invitrogen) and every twenty-four amplicons with different barcodes were mixed equally. The pooled DNA product was used to construct the Illumina paired-end library following the Illumina genomic DNA library preparation procedure. The amplicon library was paired-end sequenced (2 × 250) on an Illumina platform (Shanghai BIOZERON Biotech Co., Ltd.), according to standard protocols.

### 2.6 Statistical Analysis

Unless otherwise indicated, statistical analyses were performed using the GraphPad Prism software. All experimental data are expressed as mean ± SEM, and One-way ANOVA was used to confirm the significance between the three groups, and t-test was used to confirm the significance between the two groups. Statistical significance was set at *p* < 0.05.

## 3 Results

### 3.1 Preventive Effect of Qingchang Wenzhong Decoction on Azoxymethane/Dextran Sodium Sulfate Mice

To evaluate the preventive effect of QCWZD on colon tumors, we constructed an AOM/DSS mouse model (Figure 1A). Animals in the vehicle group exhibited normal colon morphology. Meanwhile, animals in the AOM/DSS group exhibited large number of tumors at the end of the colorectum. In addition, animals in the high-dose QCWZD group exhibited significantly reduced average number of tumors, tumor size, and tumor load compared with those in the AOM/DSS group (Figures 1B,C).

H&E staining revealed that the colorectal epithelial cells in the vehicle group were closely arranged; the recess was normal; the intestinal mucosa and submucosa were complete; and there was no inflammatory cell infiltration. However, in the AOM/DSS group, H&E staining revealed the following: local necrosis and abscission of intestinal mucosal epithelial cells; fibrous tissue hyperplasia and focal infiltration of inflammatory cells in the mucosal layer; disappearance of some intestinal glands; and fibrous connective tissue hyperplasia, as well as increased tissue space, in the submucosa. Compared with the AOM/DSS group, the QCWZD treatment groups exhibited decreased degree of inflammatory cell infiltration and more normal recess cells (Figure 1D). These results confirmed that QCWZD reduced the occurrence of AOM/DSS-induced colon tumors.

### 3.2 Qingchang Wenzhong Decoction Changed the Overall Composition of the Intestinal Microbiota

**FIGURE 2:**
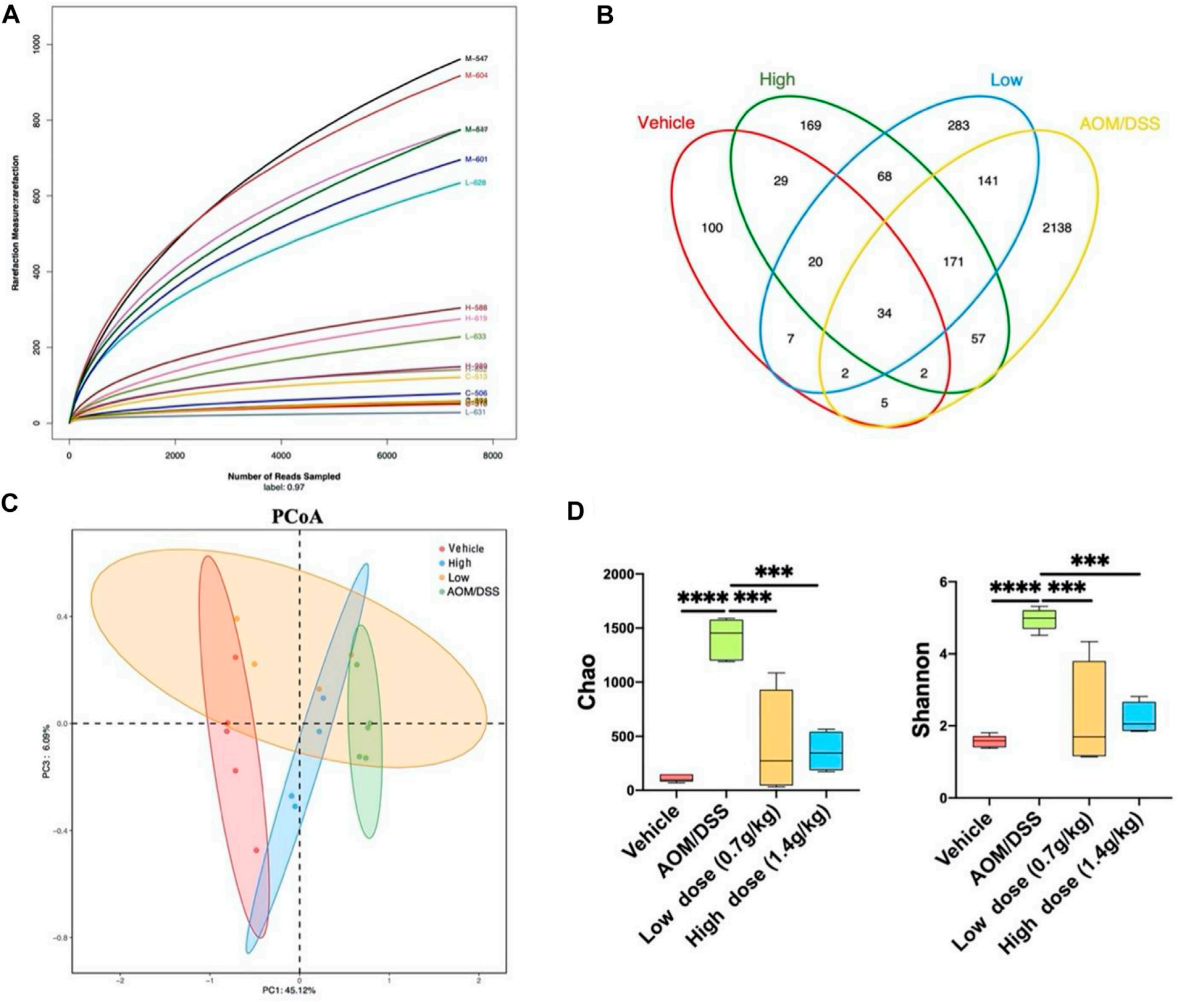
QCWZD can change the structure of intestinal microbiota in AOM/DSS mice. **(A)** Rarefaction curves determined at the 97% similarity level. **(B)** Venn diagram of OTUs in the four groups (vehicle group, AOM/DSS group, low-dose group, high-dose group). **(C)** Multiple-sample PCoA analysis. **(D)** Chao and Shannon indices. Low dose: 0.7 g/kg, High dose: 1.4 g/kg ^***^ *p* < 0.001, ^****^ *p* < 0.0001.

To determine whether QCWZD has a regulatory effect on the intestinal microbiota, we used 16S rRNA gene sequencing for operational taxonomic unit (OTU) cluster and species taxonomy analyses. Using OTU cluster analysis, we analyzed the overall structural changes in the intestinal microbiota of AOM/DSS mice after QCWZD treatment. As shown in Figure 2A, each curve was flat, indicating that the amount of sequencing data was reasonable and most OTUs were present in all samples. In addition, the Venn diagram shows that 34 OTUs coexisted in the four groups. We identified 43 OTUs in the vehicle and AOM/DSS groups, 348 OTUs in the AOM/DSS and low-dose groups, 264 OTUs in the AOM/DSS and high-dose groups, 60 OTUs in the vehicle and low-dose groups, 85 OTUs in the vehicle and high-dose groups, and 293 OTUs in the high- and low-dose groups (Figure 2B). Principal coordinates analysis (PCoA) revealed that the intestinal microbiota of the vehicle group significantly differed from that of the AOM/DSS group. Meanwhile, the distance between the QCWZD and vehicle groups was less than that of the AOM/DSS and vehicle groups (Figure 2C). Shannon and Chao indices were used to reflect the sample α-diversity index, which revealed that QCWZD changed the α-diversity of the intestinal microbiota (Figure 2D).

### 3.3 Qingchang Wenzhong Decoction Regulated the Proliferation of Bacteria in Azoxymethane/Dextran Sodium Sulfate Mice

**FIGURE 3:**
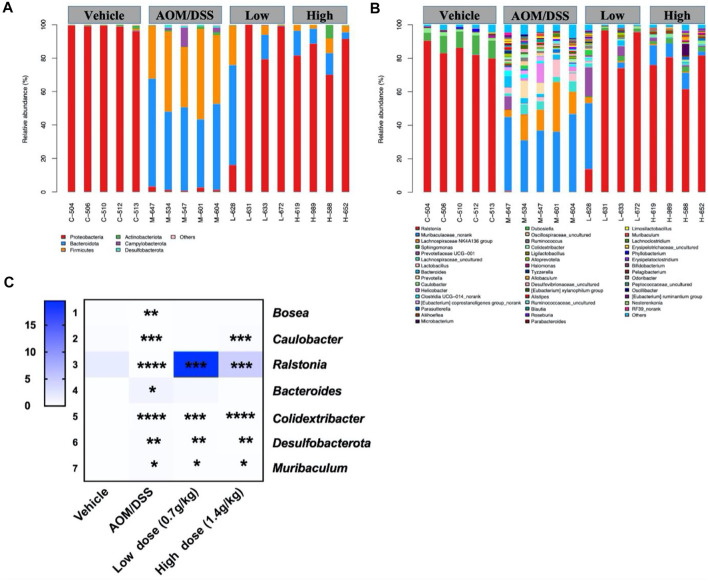
Gut microbial community structure in AOM/DSS mice after QCWZD treatment. **(A)** Microbial community bar plot according to phylum. **(B)** Microbial community bar plot according to genus. **(C)** Heatmap of gut microbiota abundance at the phylum and genus levels. Low dose: 0.7 g/kg, High dose: 1.4 g/kg ^*^ *p* < 0.05, ^**^ *p* < 0.01, ^***^ *p* < 0.001, ^****^ *p* < 0.0001.

The histogram reflected that all samples contained the phyla *Bacteroidetes*, *Firmicutes*, *Proteobacteria*, *Actinobacteria*, *Campylobacterota*, and *Desulfobacterota* (Figure 3A); the most abundant were *Bacteroidetes*, *Firmicutes*, and *Proteobacteria*. On the other hand, 46 genera were identified in all samples, mainly including *Ralstonia*, *Muribaculum*, *and* Lanchnospiraceae *NK4A136* (Figure 3B). Seven different bacteria were screened at the phylum and genus levels. At the phylum level, we found that *Desulfobacterota* existed only in the model group. At the genus level, compared with that of the model group, the abundance of *Bacteroides*, *Colidextribacter*, and *Muribaculum* in the QCWZD group significantly decreased, while the abundance of *Bosea*, *Caulobacter*, and *Ralstonia* significantly increased (Figure 3C). These results showed that QCWZD can change the composition of the intestinal microbiota and prevent the occurrence of colon tumors by decreasing the abundance of the harmful bacteria *Desulfobacterota*, *Bacteroides*, and *Muribaculum* and increasing the abundance of the beneficial bacterium *Ralstonia.*

### 3.4 Qingchang Wenzhong Decoction Prevented the Occurrence of Colonic Tumors by Improving the Function of the Intestinal Barrier and Inhibiting GSDME-Mediated Pyroptosis

**FIGURE 4:**
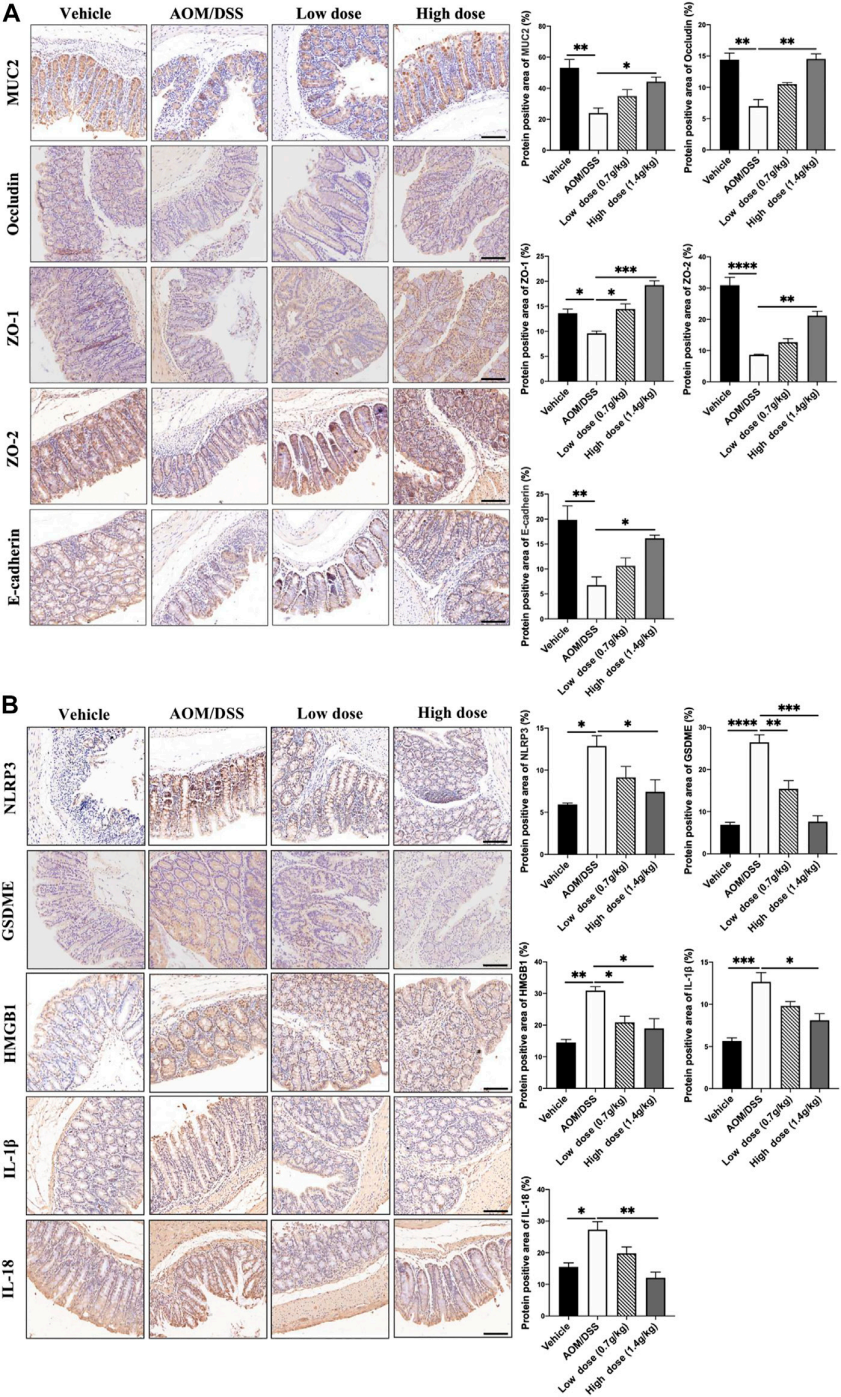
Effects of QCWZD on the intestinal barrier and pyroptosis in AOM/DSS-treated mice. **(A)** Levels of MUC2, occludin, ZO-1, ZO-2, and E-cadherin in AOM/DSS-treated mice (magnification: ×200). **(B)** Levels of *NLRP3*, GSDME, HMGB1, IL-1β, and IL-18 in AOM/DSS-treated mice (magnification: ×200). Low dose: 0.7 g/kg, High dose: 1.4 g/kg ^*^ *p* < 0.05; ^**^ *p* < 0.01; ^***^ *p* < 0.001; ^****^ *p* < 0.0001.

Under normal circumstances, microorganisms in the microbiota interact to form a dynamic balance and jointly maintain the intestinal microenvironment. Increase in the number of pathogenic bacteria and decrease in the number of probiotics will lead to intestinal microbiota imbalance and destroy the intestinal barrier (Tan et al., 2020). Therefore, using IHC, we detected the expression levels of the mucus layer-associated protein MUC2; tight junction-associated proteins Occludin, ZO-1, and ZO-2; and adhesion junction-associated protein E-cadherin. The results showed that the levels of intestinal barrier function-related proteins in the QCWZD group were significantly higher (Figure 4A) than those in the AOM/DSS group. As the microbiota-mediated destruction of the intestinal barrier further affects the occurrence of pyroptosis and promotes the occurrence of intestinal tumors, we detected the levels of proteins related to pyroptosis. We found that QCWZD inhibited the activation of the NLRP3 inflammasome, thereby decreasing GSDME expression and subsequently reducing the release of HMGB1, IL-1β, and IL-18 (Figure 4B). These results suggested that QCWZD can prevent the development of colonic tumors by improving the intestinal barrier function and inhibiting GSDME-mediated pyroptosis.

### 3.5 Preventive Effect of Qingchang Wenzhong Decoction on Small Intestinal Tumors in Apc^min/+^ Mice

**FIGURE 5:**
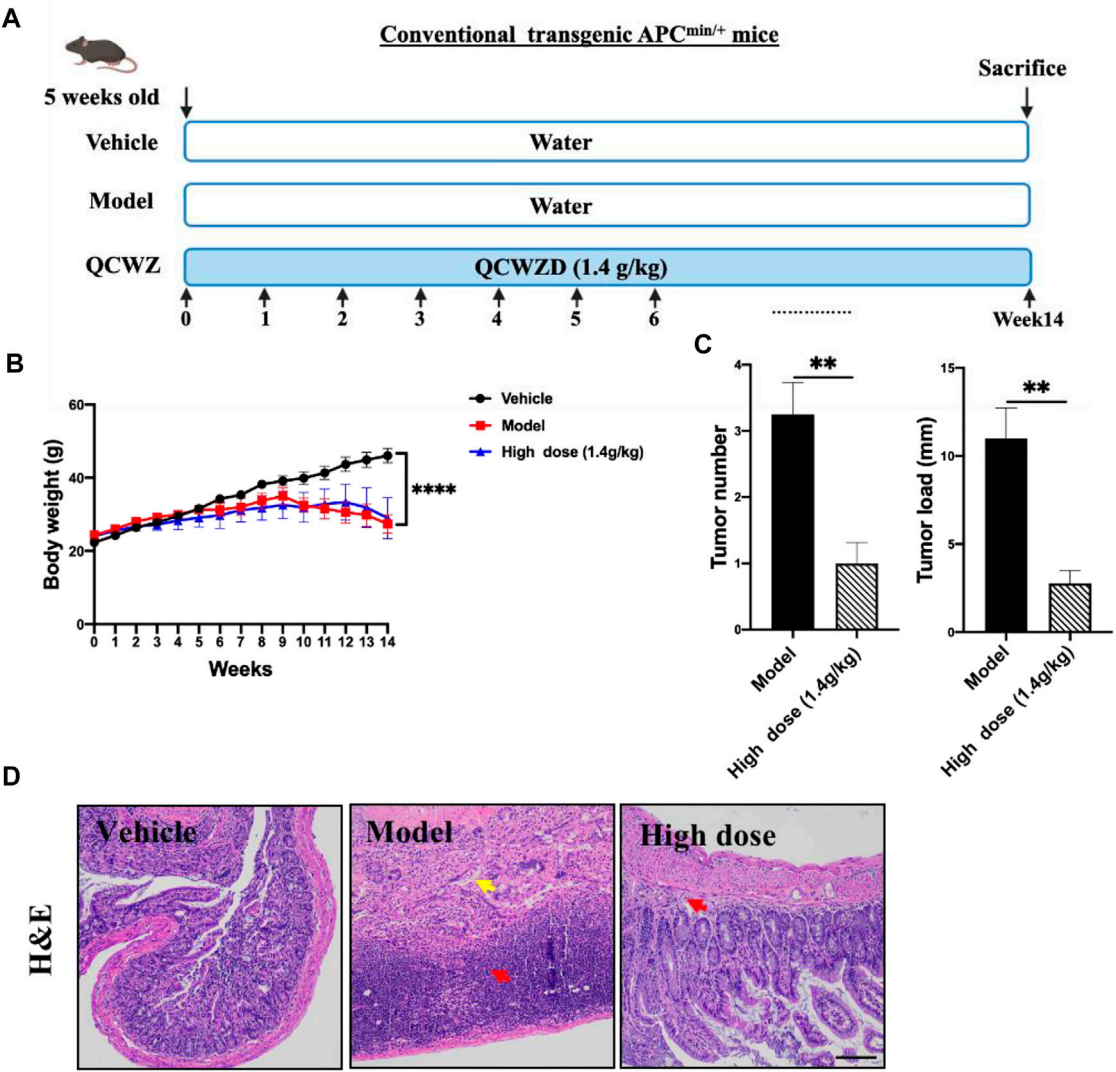
Preventive effects of QCWZD in Apc^min/+^ mice. **(A)** The Apc^min/+^ mouse model. **(B)** Measured body weight and **(C)** tumor load. **(D)** Representative H&E-stained colorectum sections (magnification: ×200). Red arrow: inflammatory cell infiltration, yellow arrow: lymphocytic infiltration; High dose: 1.4 g/kg, ^**^ *p* < 0.01, ^****^ *p* < 0.0001.

To evaluate the preventive effect of QCWZD on small intestinal tumors, we used the Apc^min/+^ mouse model (Figure 5A), an ideal intestinal tumor model. Under a high-fat diet, the Apc^min/+^ mice have obvious intestinal adenomas mostly found in the ileum and jejunum. The weight change diagram shows that compared with that of the vehicle group, the weight of the mice in the model group significantly decreased. Meanwhile, after high-dose QCWZD treatment, no significant difference was noted between the weight of the mice in the high-dose QCWZD and model groups (Figure 5B). However, high-dose QCWZD reduced the tumor number and load in Apc^min/+^ mice (Figure 5C). In addition, H&E staining showed that compared to that of the vehicle group, the intestinal tissue structure of the model group was severely abnormal, and a large amount of inflammatory cell infiltration was observed in the tissue. After high-dose QCWZD treatment, the intestinal tissue structure was slightly abnormal, and the degree of inflammatory cell infiltration decreased (Figure 5D). These data showed that QCWZD can prevent the occurrence of small intestinal tumors.

### 3.6 Qingchang Wenzhong Decoction Changed the Overall Composition of the Intestinal Microbiota in Apc^min/+^ Mice

**FIGURE 6:**
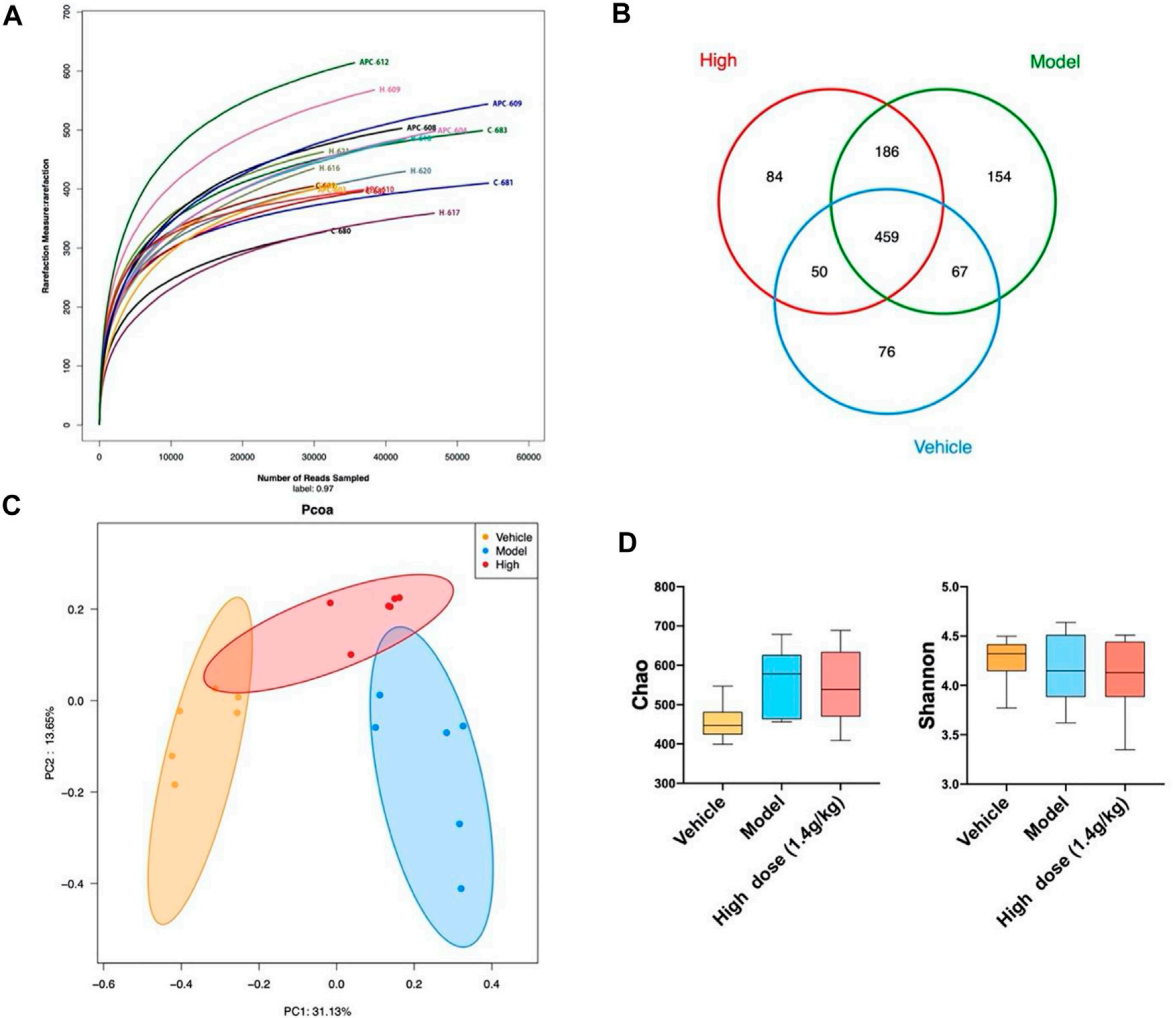
QCWZD can change the structure of intestinal microbiota in Apc^min/+^ mice. **(A)** Rarefaction curves determined at the 97% similarity level. **(B)** Venn diagram of OTUs in the three groups (vehicle group, model group, high-dose group). **(C)** Multiple-sample PCoA analysis. **(D)** Chao and Shannon indices. High dose: 1.4 g/kg.

Considering that QCWZD can change the overall intestinal microbiota composition of AOM/DSS mice, we investigated whether the same effect can be observed on Apc^min/+^ mice. We also used 16S rRNA gene sequencing to analyze changes in the overall intestinal microbiota of the mice. The rarefaction curves showed that the species richness in the different samples significantly varied; the rarefaction curve of each sample was flat, indicating that the amount of sequencing data was reasonable (Figure 6A). Based on the sequencing data of these samples and the Venn diagram, 459 OTUs were identified in the four groups, 526 OTUs in the vehicle and model groups, 645 OTUs in the model and high-dose groups, and 509 OTUs in the vehicle and high-dose groups (Figure 6B). The PCoA diagram shows that the intestinal microbiota of the vehicle and model groups are significantly different. After high-dose QCWZD treatment, the intestinal microbiota of the high-dose group became similar to that of the vehicle group (Figure 6C). In addition, no significant difference was noted in the Chao and Shannon indices (Figure 6D). Overall, QCWZD can prevent the occurrence of small intestinal tumors by altering the overall composition of the intestinal microbiota in Apc^min/+^ mice.

### 3.7 Qingchang Wenzhong Decoction Regulated the Proliferation of Bacteria in Apc^min/+^ Mice

**FIGURE 7:**
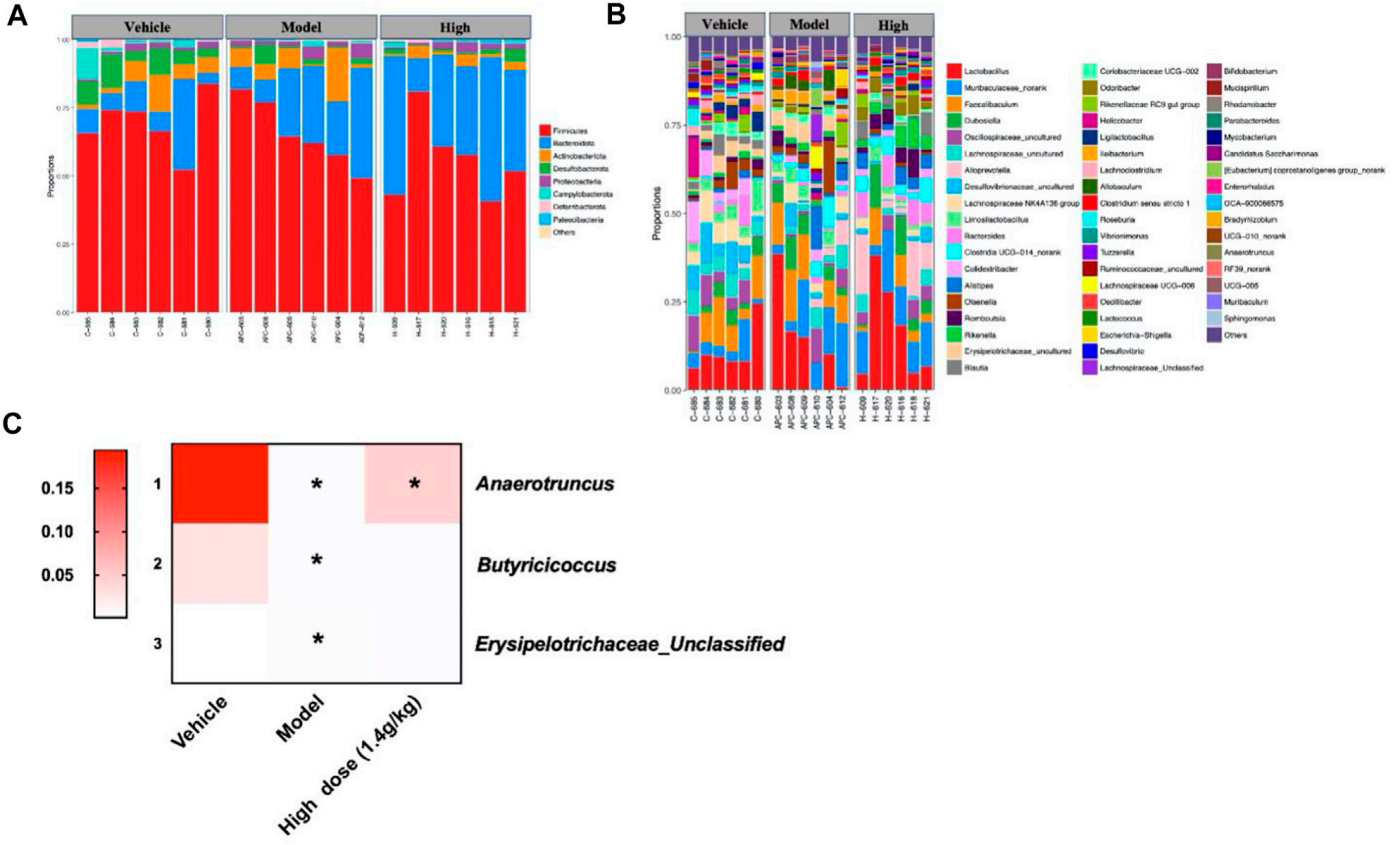
Gut microbial community structure in Apc^min/+^ mice after QCWZD treatment. **(A)** Microbial community bar plot according to phylum. **(B)** Microbial community bar plot according to genus. **(C)** Heatmap of gut microbiota abundance at the phylum and genus levels. High dose: 1.4 g/kg, ^*^ *p* < 0.05.

Since previous studies have shown that QCWZD can regulate the specific intestinal microbiota of AOM/DSS mice, we also used 16S rRNA to analyze the intestinal microbiota structure of Apc^min/+^ mice at the phylum and genus levels. The phylum level mainly included *Firmicutes*, *Bacteroidetes*, *Actinobacteria*, *Desulfobacterota*, *Proteobacteria*, *Campylobacterota*, *Verrucomicrobiota*, *Deferibacterota*, and *Paesciabacteria*, of which *Firmicutes* and *Bacteroidota* accounted for a large proportion (Figure 7A). The genus level included *Lactobacillus*, Muribaculaceae*_norank*, and *Faecalibaculum* (Figure 7B); 56 genera were identified in all samples. In addition, three different bacteria were screened at the phylum and genus levels according to the structural distribution of the intestinal microbiota in each group. Compared to that of the vehicle group, the abundance of *Desulfobacterota*, *Anaerotruncus*, and *Butyricoccus* in the model group significantly decreased. Albeit insignificantly, high-dose QCWZD changed this trend. Further, the abundance of Erysipelotrichaceae*_unclassified* was high in the model group and low in the vehicle and high-dose QCWZD groups (Figure 7C). These data indicated that QCWZD reduced the abundance of the harmful bacteria Erysipelotrichaceae*_ unclassified* and increased the abundance of the beneficial bacteria *Anaerotruncus* and *Butyricoccus* to prevent the occurrence of small intestinal tumors.

### 3.8 Qingchang Wenzhong Decoction Prevented the Occurrence of Small Intestinal Tumors by Improving the Function of the Intestinal Barrier and Inhibiting GSDME-Mediated Pyroptosis

**FIGURE 8:**
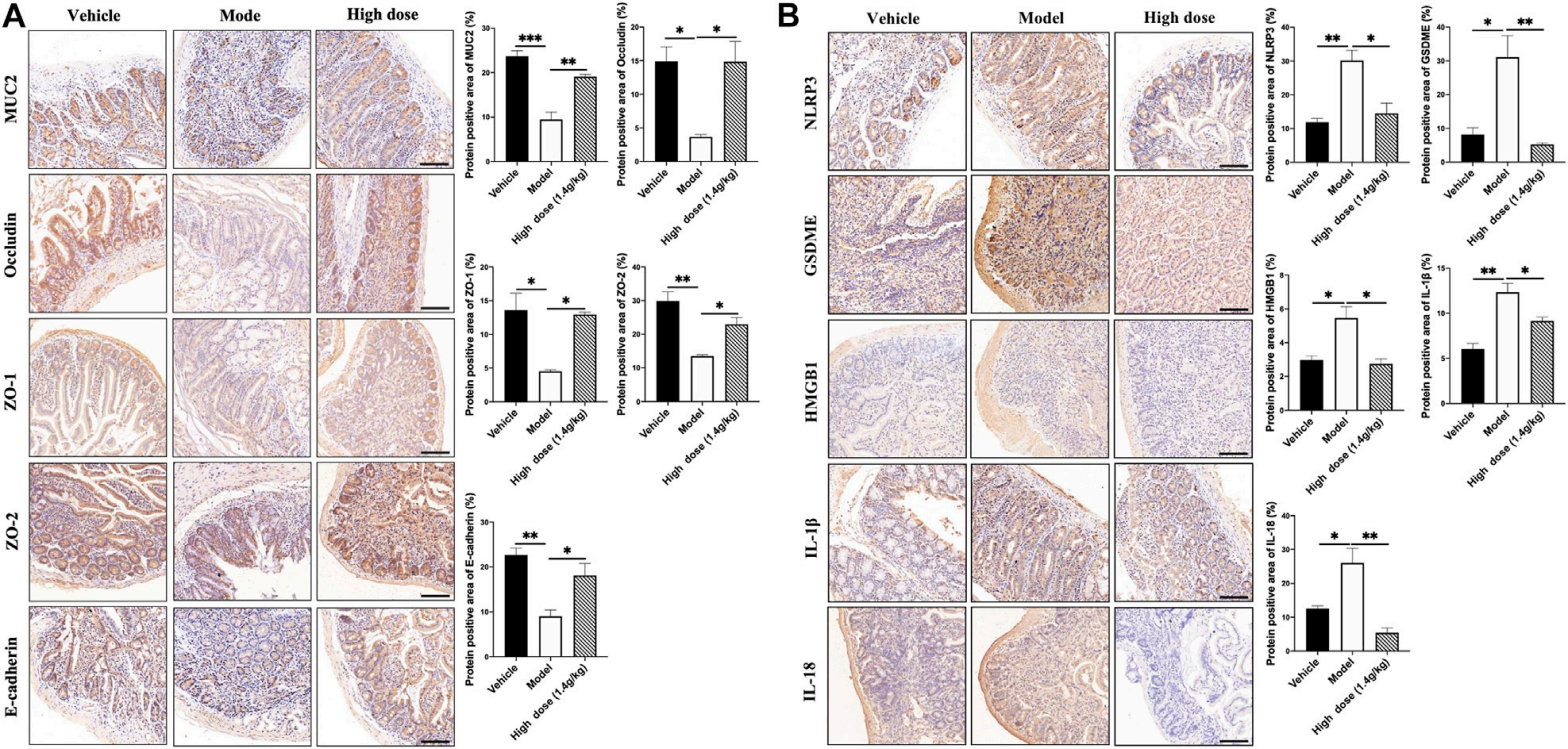
Effects of QCWZD on the intestinal barrier and pyroptosis in Apc^min/+^ mice. **(A)** Levels of MUC2, occludin, ZO-1, ZO-2, and E-cadherin in Apc^min/+^ mice (magnification: ×200). **(B)** Levels of *NLRP3*, GSDME, HMGB1, IL-1β, and IL-18 in Apc^min/+^ mice (magnification: ×200). High dose: 1.4 g/kg, ^*^ *p* < 0.05, ^**^ *p* < 0.01.

In AOM/DSS mice, QCWZD reduced the occurrence of colonic tumors by improving the function of the intestinal barrier and inhibiting GSDME-mediated pyroptosis. Using IHC, we analyzed the expression levels of intestinal barrier function-related proteins in the small intestine to verify whether the intestinal barrier function improved in the Apc^min/+^ mouse model. As shown in Figure 8A, the mucus layer-associated protein MUC2, tight junction-associated proteins Occludin, ZO-1, and ZO-2, and adhesion junction-associated protein E-cadherin were poorly expressed in the model group; however, their expression significantly increased after QCWZD treatment. These results suggested that QCWZD improved the intestinal barrier function. At the same time, pyroptosis-associated proteins were also detected, showing that QCWZD can prevent the development of small intestinal tumors in the Apc^min/+^ model by inhibiting GSDME-mediated pyroptosis (Figure 8B).

## 4 Discussion

The number of microorganisms in an adult intestine is as high as 10^14^, which is ten times the total number of human cells (Tremaroli and Backhed, 2012). Under normal circumstances, various microbiota restrict and depend on each other to maintain dynamic balance and jointly maintain the intestinal microenvironment. Once this balance is destroyed, the number of pathogenic bacteria increases and the number of probiotics decreases, leading to intestinal microbiota imbalance and subsequent onset of intestinal diseases. Recent studies have shown that the intestinal microbiota contributes to the pathogenesis and prognosis of intestinal tumors (Geller et al., 2017; Fan et al., 2018; Zheng et al., 2018). However, the complex interaction between the intestinal microbiota and intestinal tumors is still the main obstacle for the prevention and treatment of intestinal tumors.

In this study, we used AOM/DSS and Apc^min/+^ mouse models to study the effect of QCWZD on intestinal tumors. The results revealed that QCWZD exerted a pronounced protective effect against intestinal tumors, which was manifested by reducing the number of colonic tumors, tumor size and load, and degree of colonic lesions in AOM/DSS mice. QCWZD also decreased the small intestinal tumor load of Apc^min/+^ mice.

The intestinal microbiota, including symbiotic bacteria, probiotics, and pathogens, play an important role in human health. We used 16S rRNA gene sequencing to analyze abundance, structural distribution, and intergroup differences in the microbial community. In AOM/DSS mice, seven different bacteria were found at the phylum and genus levels: Desulfobacterota, *Bacteroides*, *Bosea*, *Caulobacter*, *Colidextribacter*, *Limoslactobacillus*, *Muribaculum*, and *Sphingomonas.* Desulfobacterota, *Bacteroides*, *Colidextribacter*, *Limoslactobacillus*, and *Muribaculum* were highly enriched in the model group; whereas *Bosea*, *Caulobacter*, *and Sphingomonas* were enriched in the control and QCWZD treatment groups. *Bacteroides* is a potential “driver” of CRC, and its specific bacterial components can cause DNA damage in colonic epithelial cells (Tjalsma et al., 2012). Desulfobacterota is associated with intestinal immunity and microbiota imbalance (Xiao et al., 2021). *Muribaculum* has been reported to be positively correlated with colonic proinflammatory cytokines (Reinoso Webb et al., 2018). *Roseburia* is a probiotic that can metabolize short-chain fatty acids (SCFAs), and the metabolites produced are beneficial to intestinal health (Tjalsma et al., 2012; Louis et al., 2014; Flemer et al., 2018). In Apc^min/+^ mice, three different bacteria were screened at the phylum and genus levels: *Anaerotruncus*, *Butyricoccus*, and Erysipelotrichaceae*_unclassified*. Members of Erysipelotrichaceae can aggravate intestinal inflammation (Kaakoush, 2015), while *Anaerotruncus* and *Butyricoccus* may produce SCFAs. The production of SCFAs can inhibit the occurrence of intestinal tumors (Shi et al., 2020; Fang et al., 2021). *Anaerotruncus* is considered conducive for the production of butyrate and has antiobesity effects (Polansky et al., 2015; Liu et al., 2016). In conclusion, the above results showed that QCWZD can prevent the occurrence of intestinal tumors by increasing the number of beneficial bacteria and reducing the number of harmful bacteria in the intestine. The intestinal microbiota/inflammation axis also plays an important role in the occurrence and development of intestinal tumors. Through inductive analysis, we found that the regulatory mechanism of pathogenic bacteria was closely related to inflammation and QCWZD can ameliorate the intestinal inflammatory infiltration (Supplementary Figure S1). Further, intestinal microbiota imbalance will lead to the destruction of the intestinal barrier. IHC revealed that QCWZD can reinforce the intestinal mucous layer and mechanical barrier composed of TJs and AJs.

The occurrence and development of cancer are often accompanied by serious inflammatory reactions and cell pyroptosis, a type of inflammatory cell death (Ruan et al., 2020; Zheng and Li, 2020; Ju et al., 2021). Zheng et al. (Yu et al., 2019) found that GSDME cleavage by cysteine aspartase 3 determines the pyroptosis of colon cancer cells induced by lobaplatin. The destruction of the intestinal barrier will further affect the occurrence of pyroptosis. To explore whether the preventive effect of QCWZD on intestinal tumors was related to pyroptosis, we conducted an IHC analysis. The results showed that pyroptosis-associated proteins were highly expressed in the AOM/DSS and Apc^min/+^ mouse models; however, the expression level decreased after QCWZD treatment, indicating that QCWZD inhibits the activation of the NLRP3 inflammasome. This phenomenon results in the inhibition of GSDME-mediated pyroptosis and reduced release of HMGB1, IL-1β, and IL-18.

A recent study found that QZWZD accelerates intestinal mucosal healing by modulating dysregulated gut microbiome, intestinal barrier, and immune responses in mice (Sun et al., 2021). In this study, we used a DSS mouse model to prove that QCWZD can reverse DSS-induced intestinal disorders, change the metabolic status, promote recovery from epithelial damage, reduce intestinal inflammation, and activate Wnt/β-catenin signaling, thereby improving intestinal mucosal barrier integrity. We also found that QCWZD reduced the expression of β-catenin, glycogen synthase kinase-3β, and Axin2 (Supplementary Figure S2). However, we used different models, i.e., AOM/DSS and Apc models, to explore the mechanism by which QCWZD prevents the development of intestinal tumors. Our study found that QCWZD can regulate the intestinal microbiota in a different manner from that described in the aforementioned studies. Further, considering the new perspectives on pyroptosis, we found that QCWZD can inhibit GSDME-mediated pyroptosis to prevent the occurrence of intestinal tumors.

**FIGURE 9:**
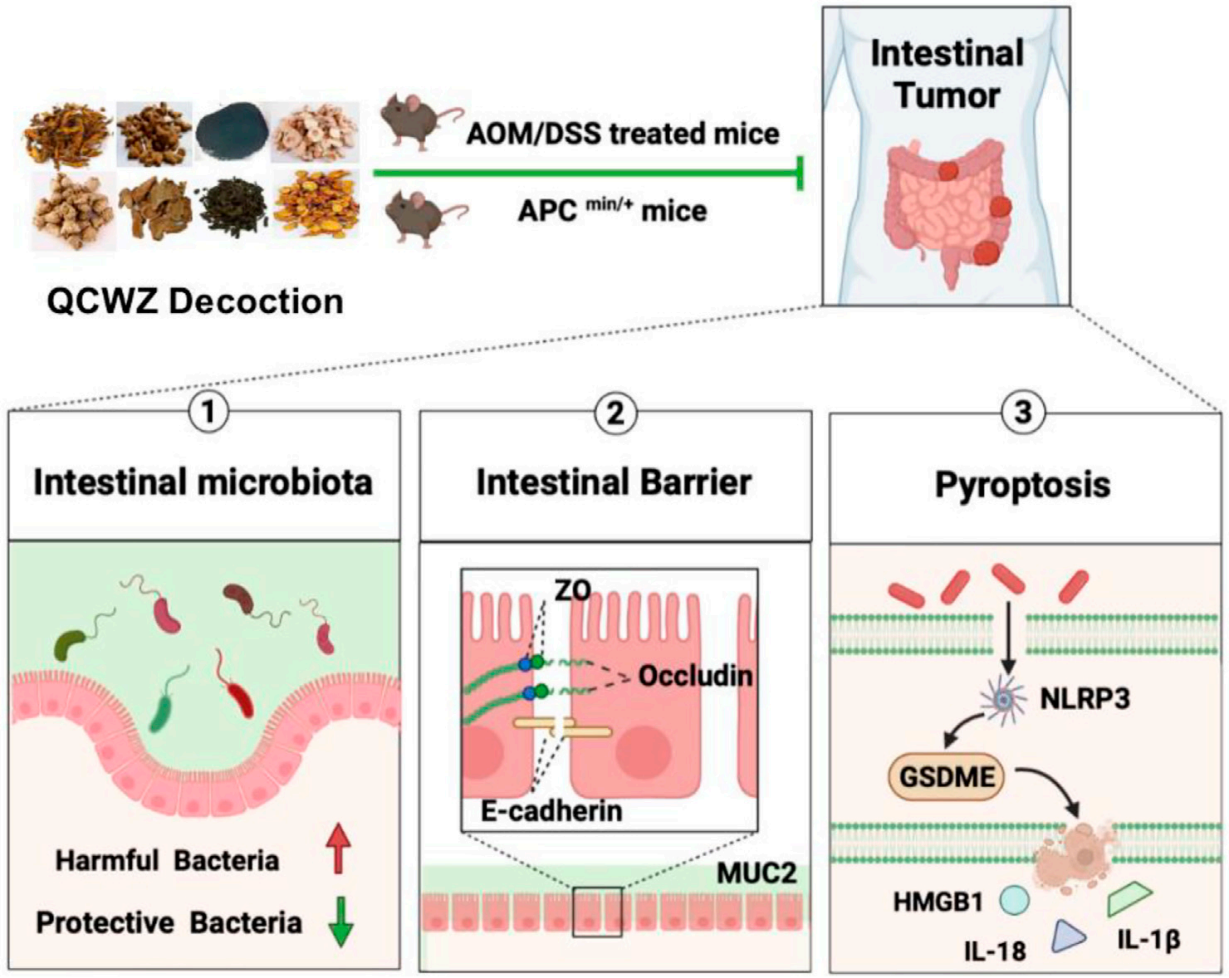
Schematic diagram of how QCWZD prevents intestinal tumorigenesis.

In conclusion, our data showed that QCWZD can effectively prevent the occurrence of intestinal tumors in AOM/DSS and Apc^min/+^ mouse models. Its protective mechanism is related to regulating the intestinal microbiota, inhibiting HMGB1 release mediated by GSDME, and improving the intestinal barrier (Figure 9). The mechanism of action of QCWZD in colitis has been previously reported; however, to the best of our knowledge, we were the first to report a preventive mechanism against intestinal tumors. Our results provided theoretical support for the clinical application of QCWZD. This study provided new ideas and helped in the secondary research and development, optimization, and innovation of traditional Chinese medicine. It broke through the limitations of research and application.

## Data Availability

The datasets presented in this study can be found in online repositories. The names of the repository/repositories and accession number(s) can be found below: https://www.ncbi.nlm.nih.gov/, PRJNA794912;https://www.ncbi.nlm.nih.gov/, PRJNA79648.

## Abbreviations

AJS, adhesive junctions; AOM, azoxymethane; CRC, colorectal cancer; DSS, dextran sulfate sodium; GSDME, gasdermin E; H&E, hematoxylin eosin; IHC, Immunohistochemistry; OTU, operational taxomonic units; QCWZD, Qingchang Wenzhong decoction; UC, ulcerative Colitis; SCFAs, short-chain fatty acids; TJs, tight junctions.
