# Supplementary material for: Qingchang Wenzhong Decoction Prevents the Occurrence of Intestinal Tumors by Regulating Intestinal Microbiota and Gasdermin E
Source: Front Physiol. 2022 Jul 14;13:917323. doi: 10.3389/fphys.2022.917323 (PMC9329543; doi:10.3389/fphys.2022.917323)

Raw images for HE/Phenotype/ IHC

Figure 1B

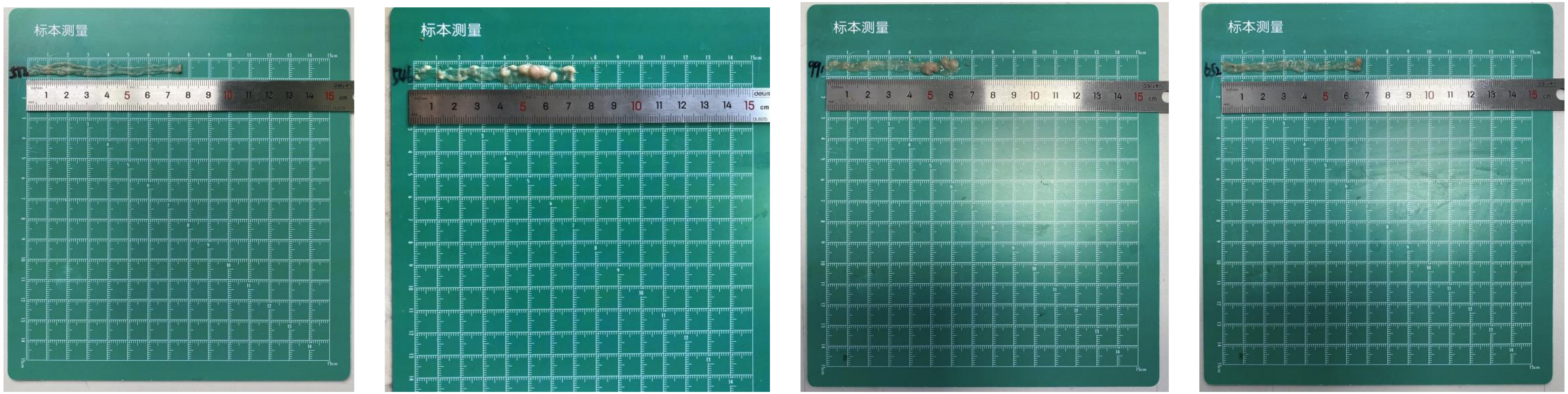

Vehicle | AOM/DSS | Low dose (0.7g/kg) | High dose (1.4g/kg)

Figure 1D

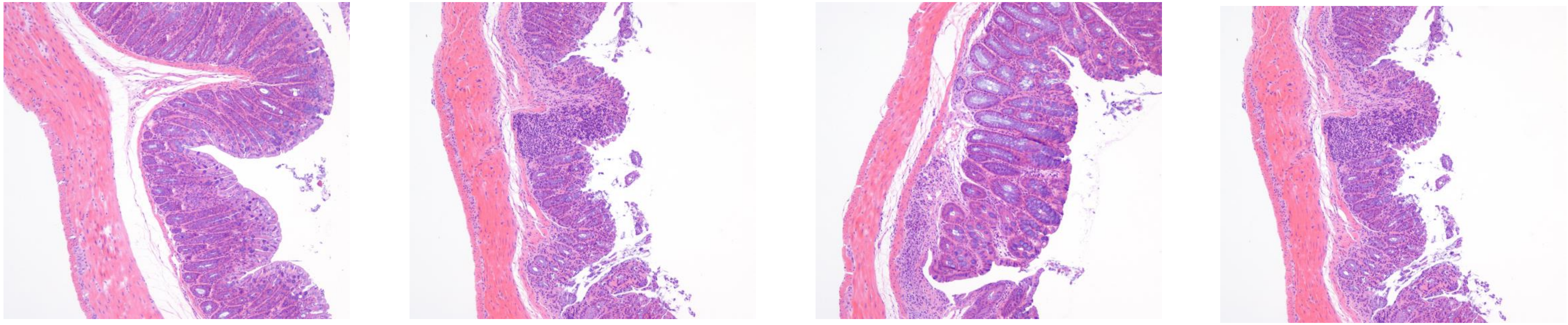

Vehicle AOM/DSS Low dose (0.7g/kg) High dose (1.4g/kg)

Figure 4A

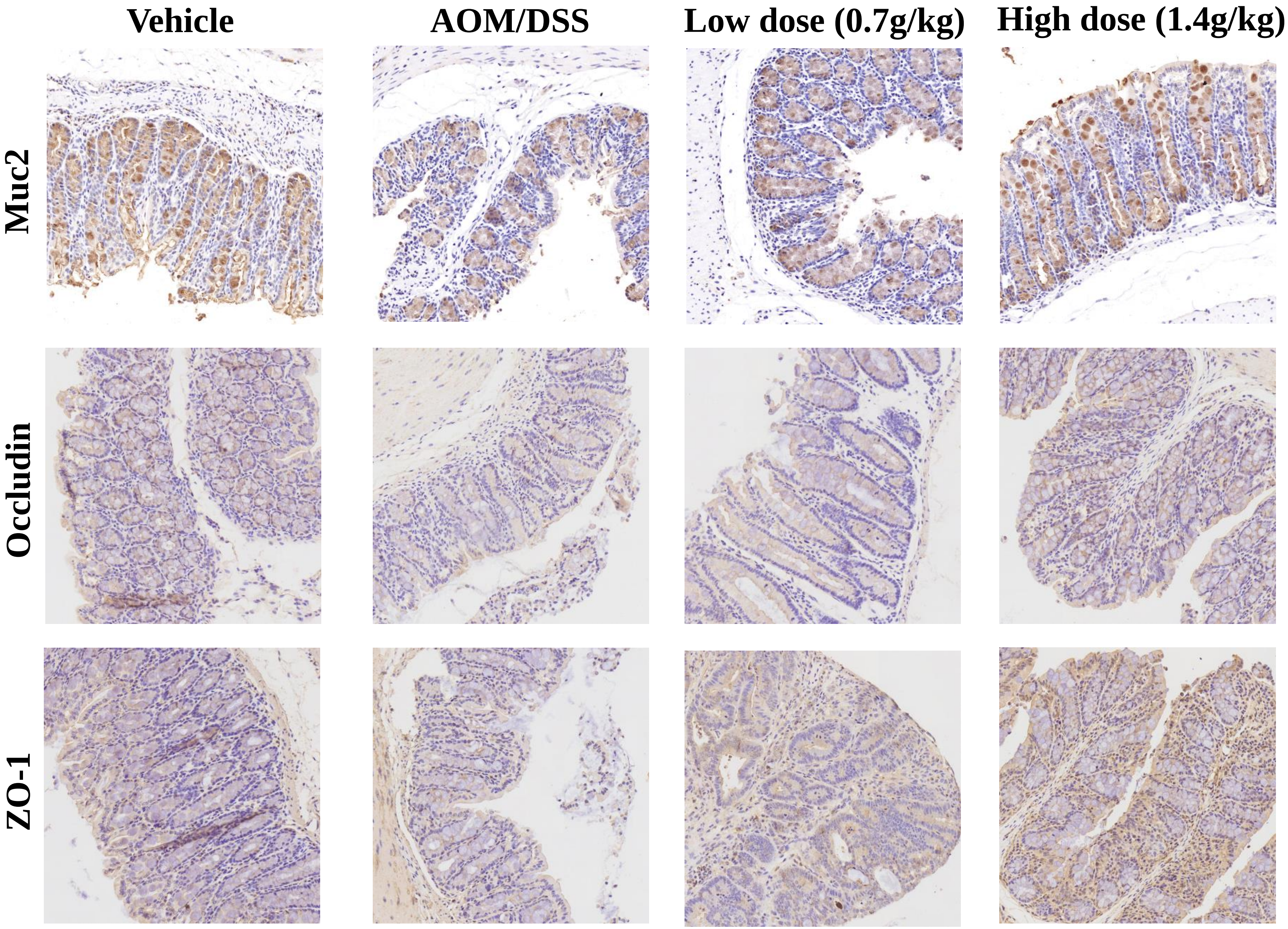
Vehicle
AOM/DSS
Low dose (0.7g/kg)
High dose (1.4g/kg)
Muc2
Occludin
ZO-1

Figure 4A

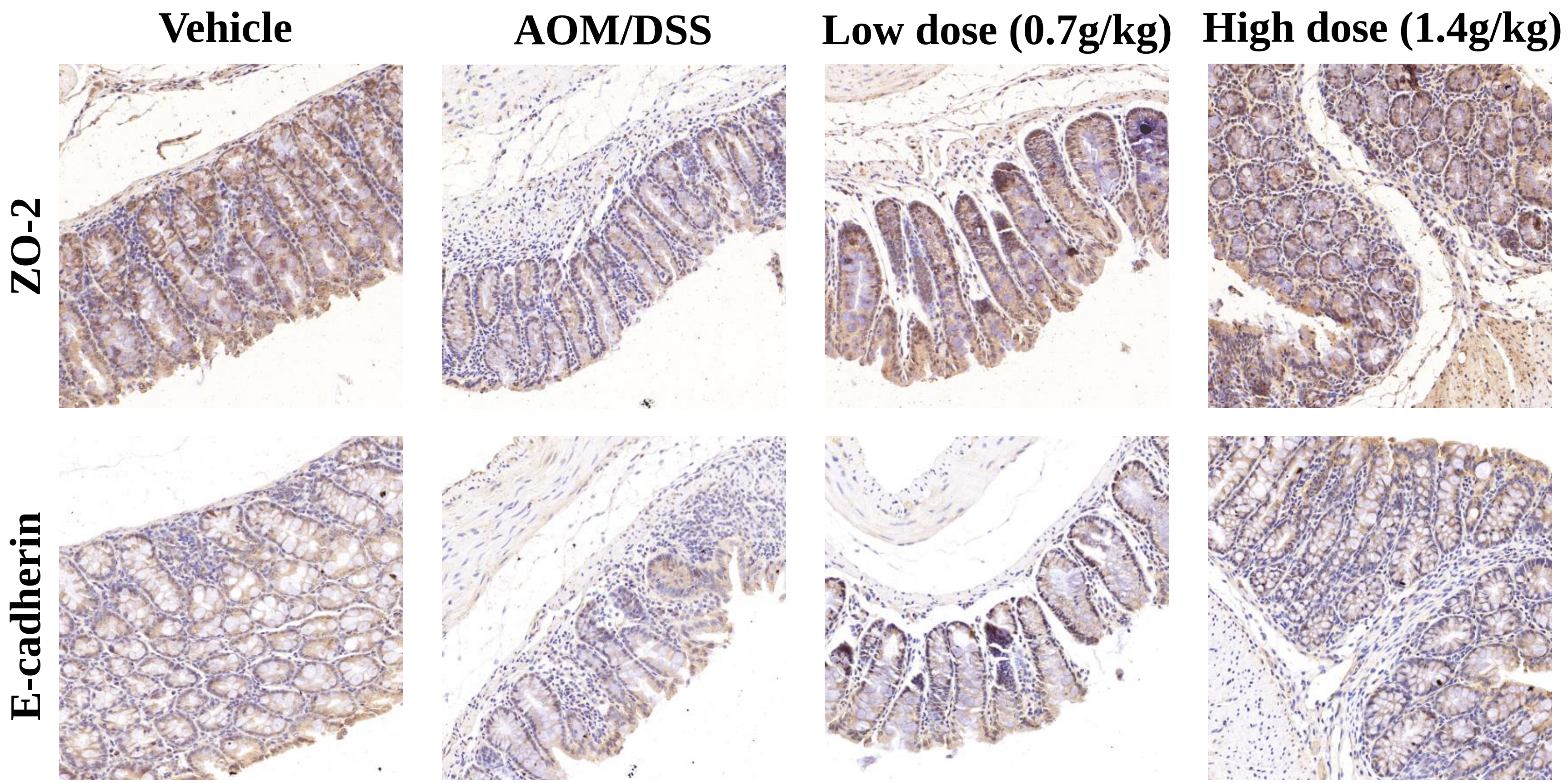

Vehicle
AOM/DSS
Low dose (0.7g/kg)
High dose (1.4g/kg)
ZO-2
E-cadherin

Figure 4B

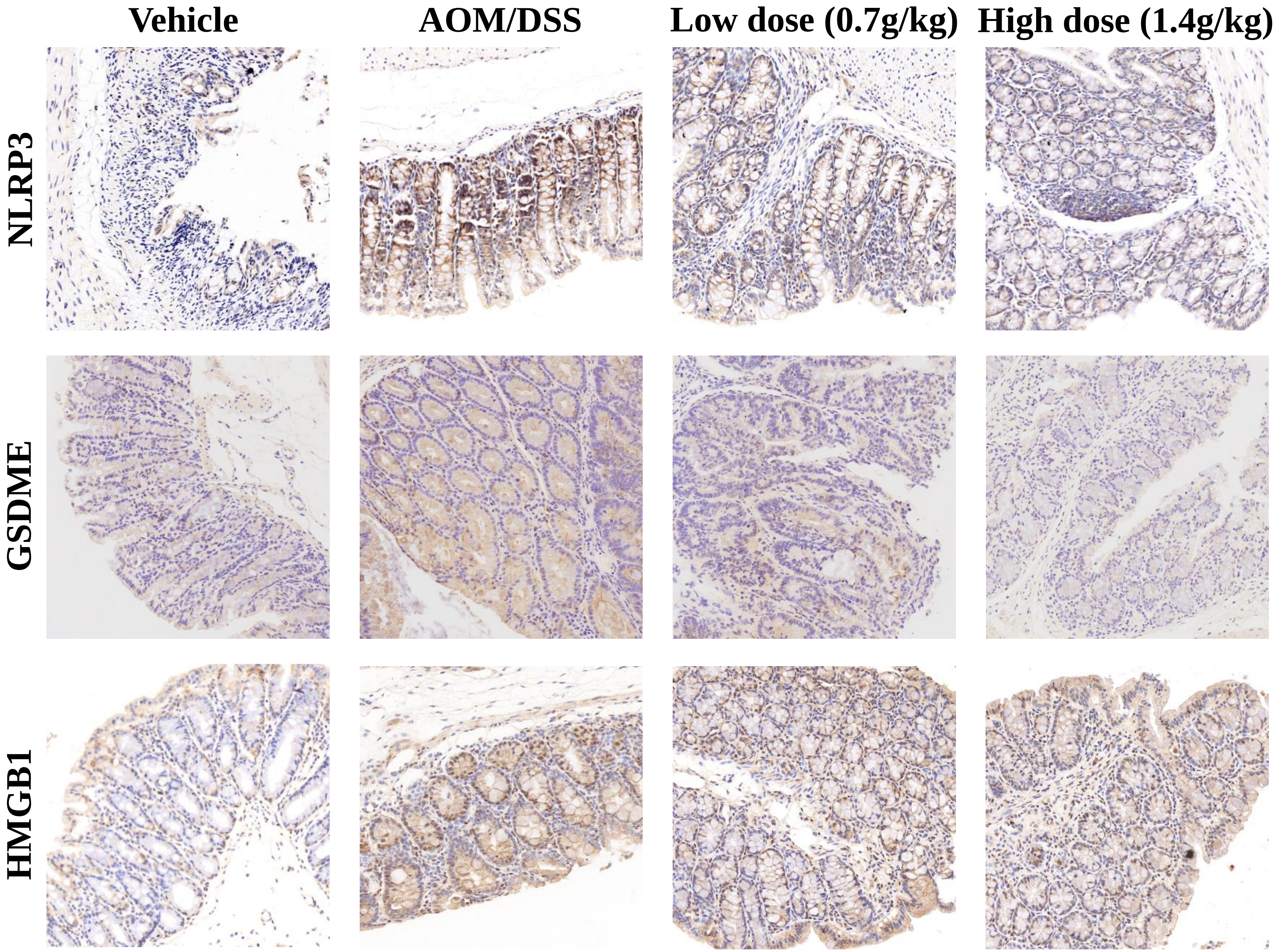

Vehicle
AOM/DSS
Low dose (0.7g/kg)
High dose (1.4g/kg)
NLRP3
GSDME
HMGB1

Figure 4B

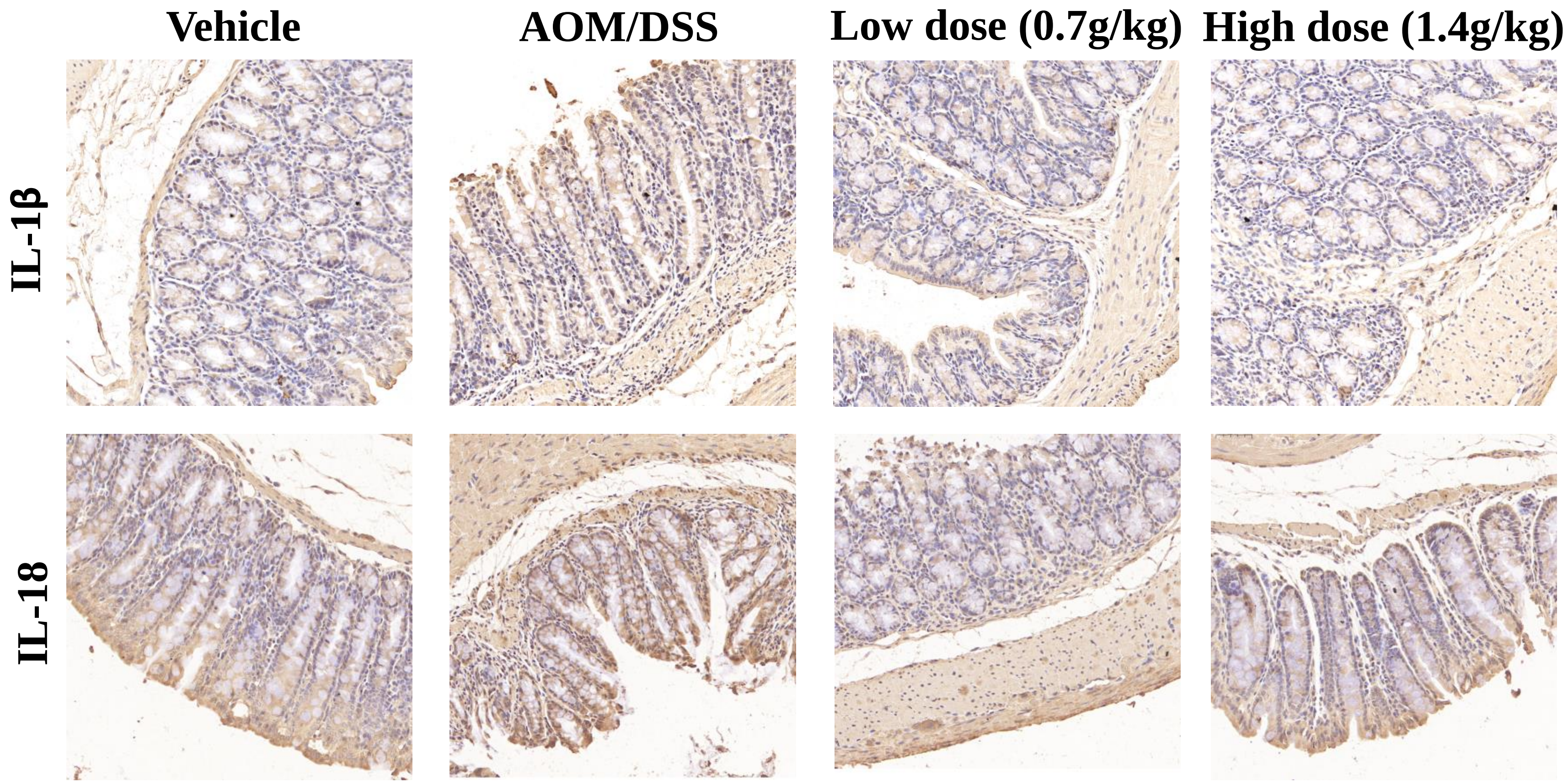

Figure 5D

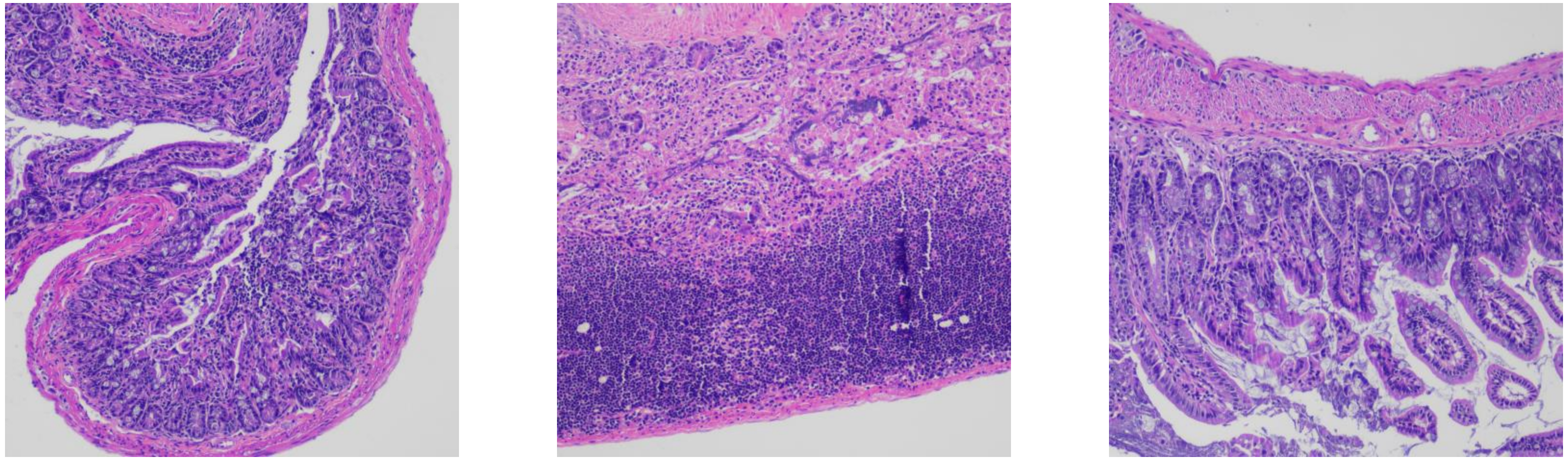

Vehicle **Model** **High dose (1.4g/kg)**

Figure 8A

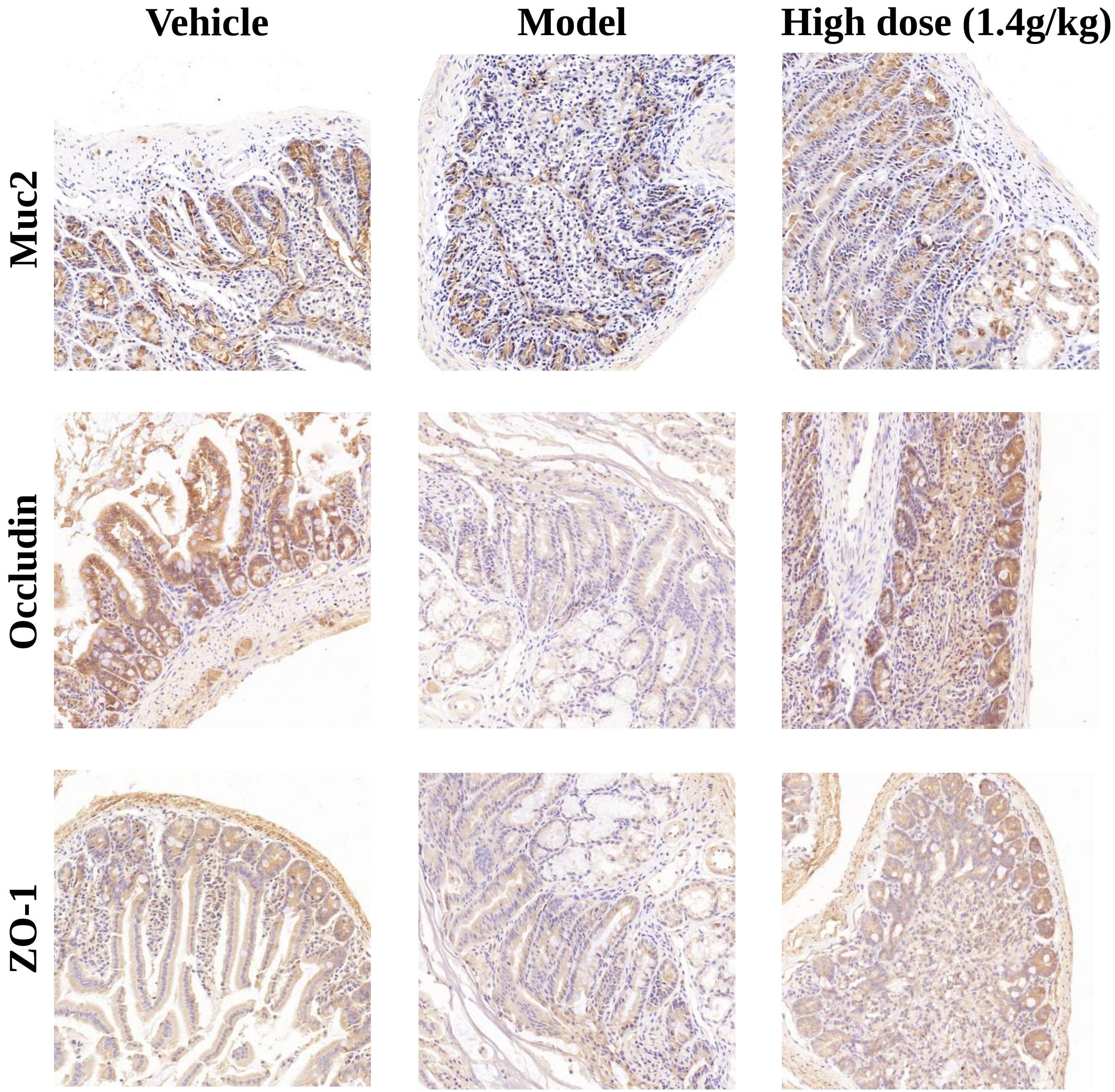

Figure 8A

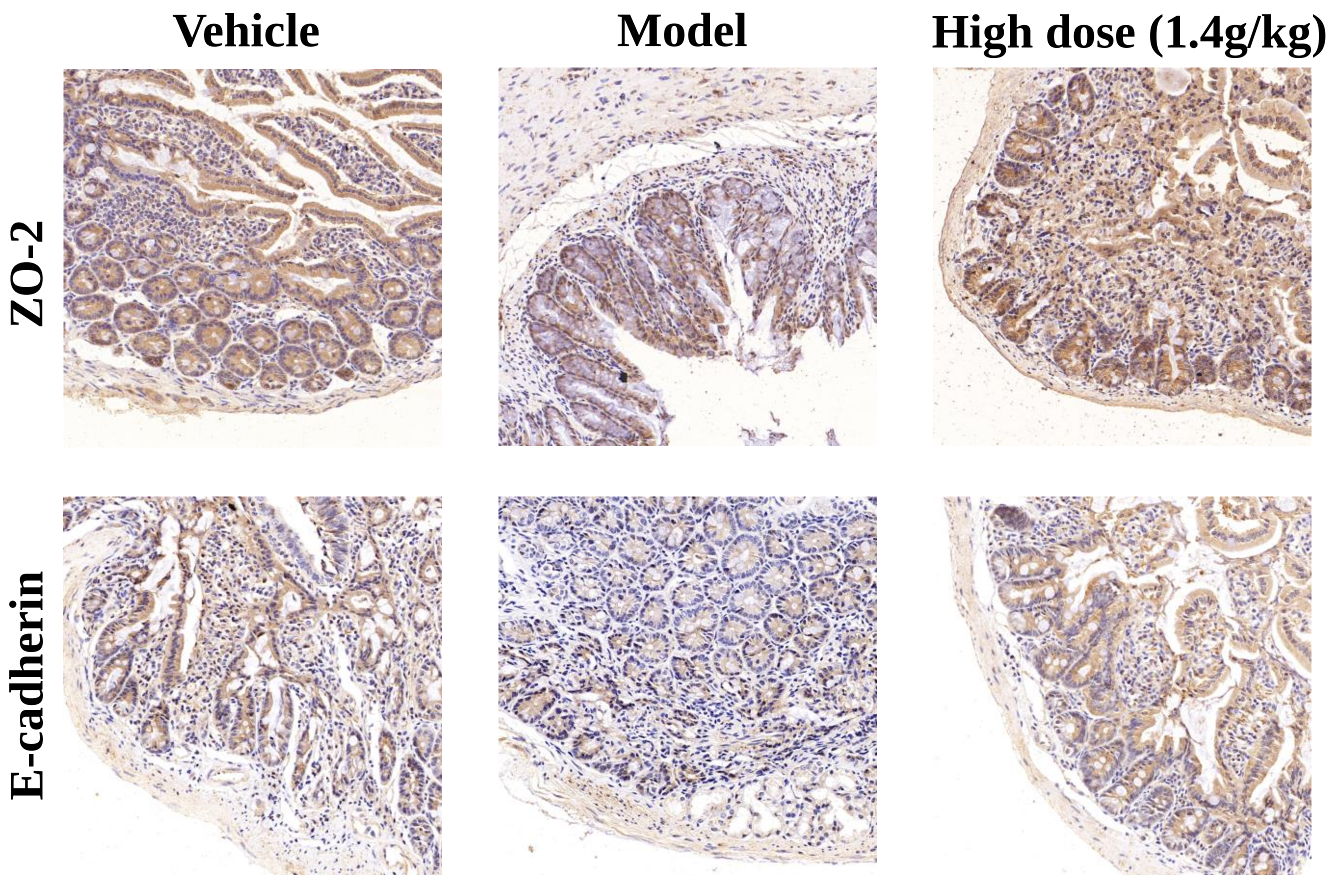

Figure 8B

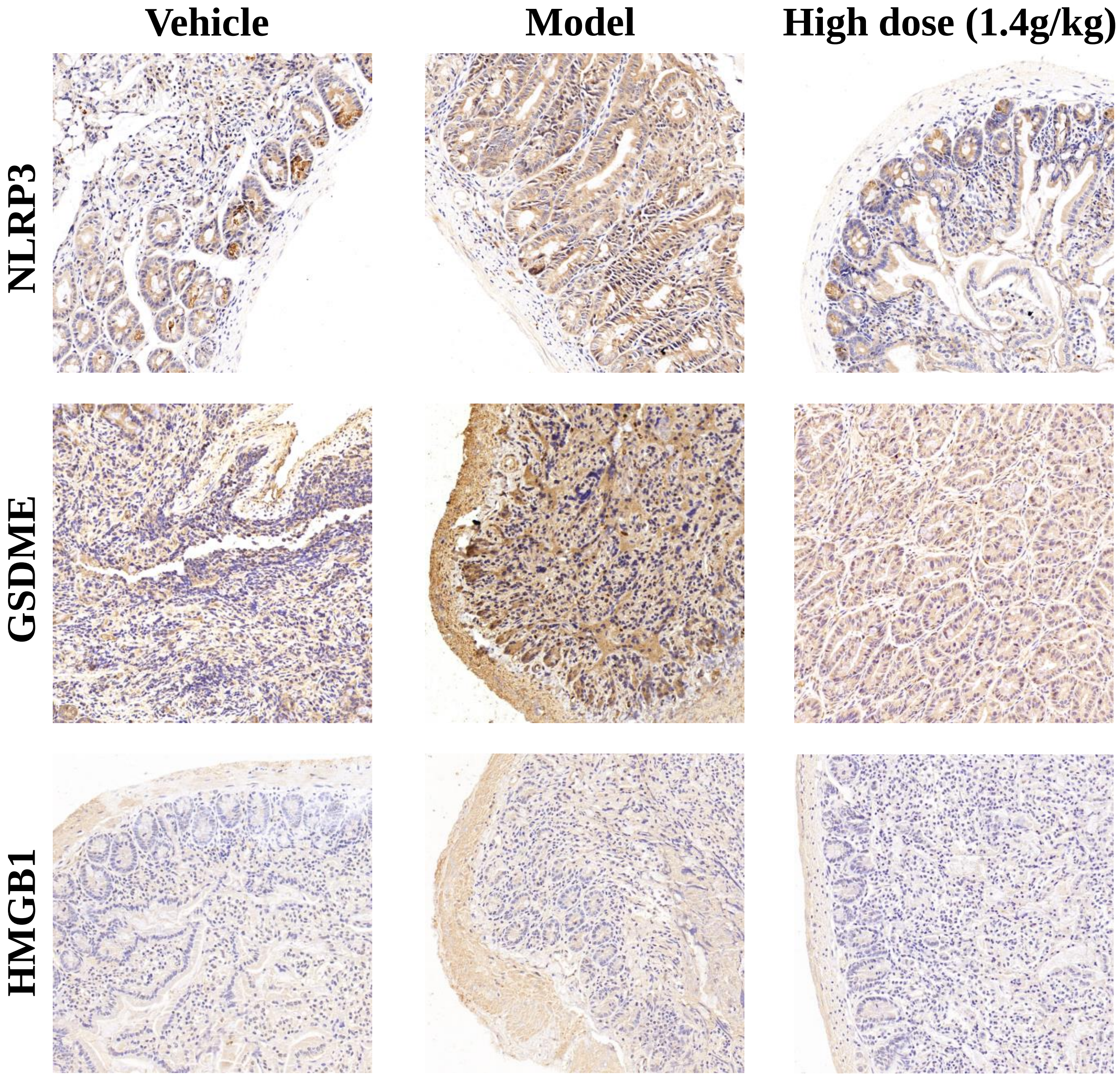

Figure 8B

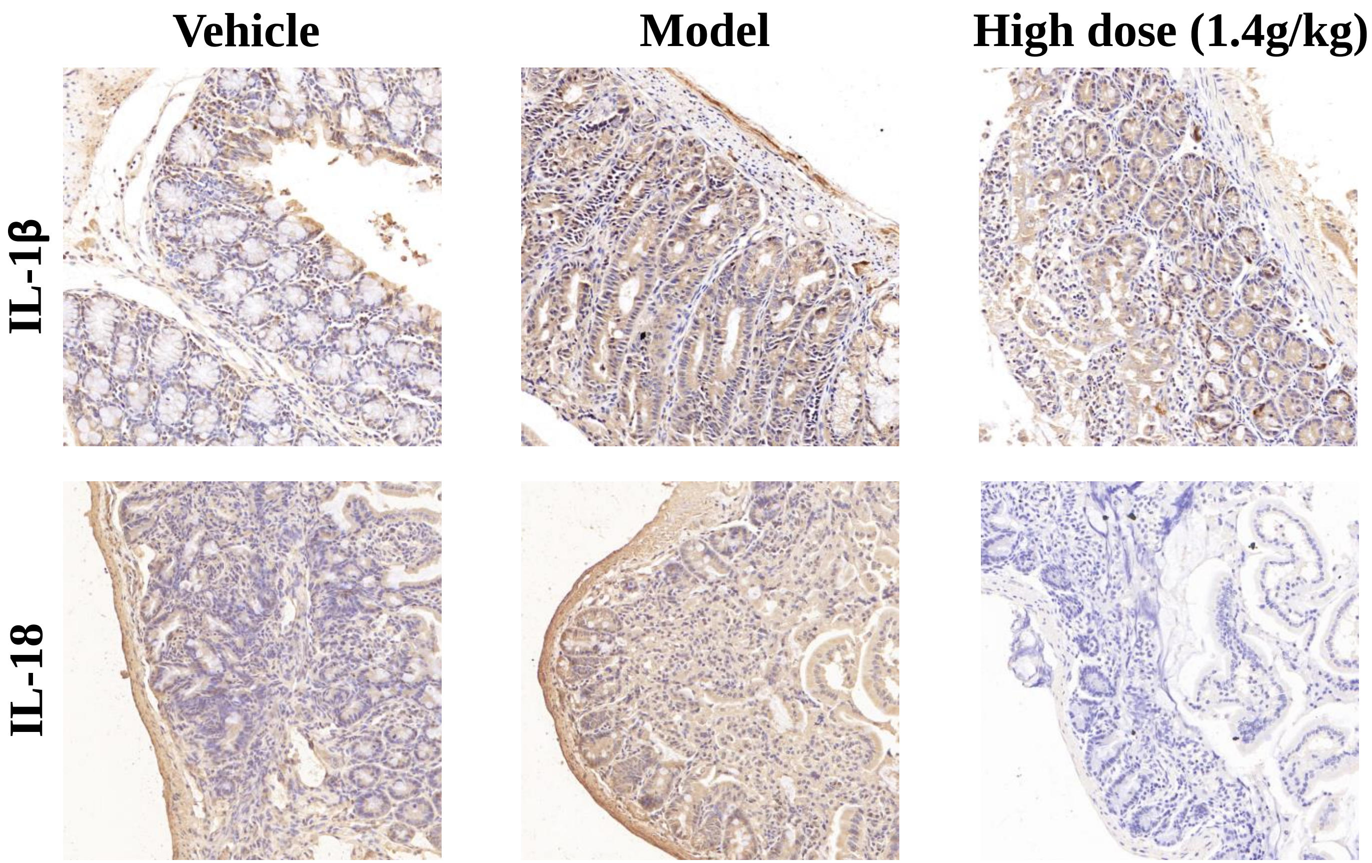

Figure S1

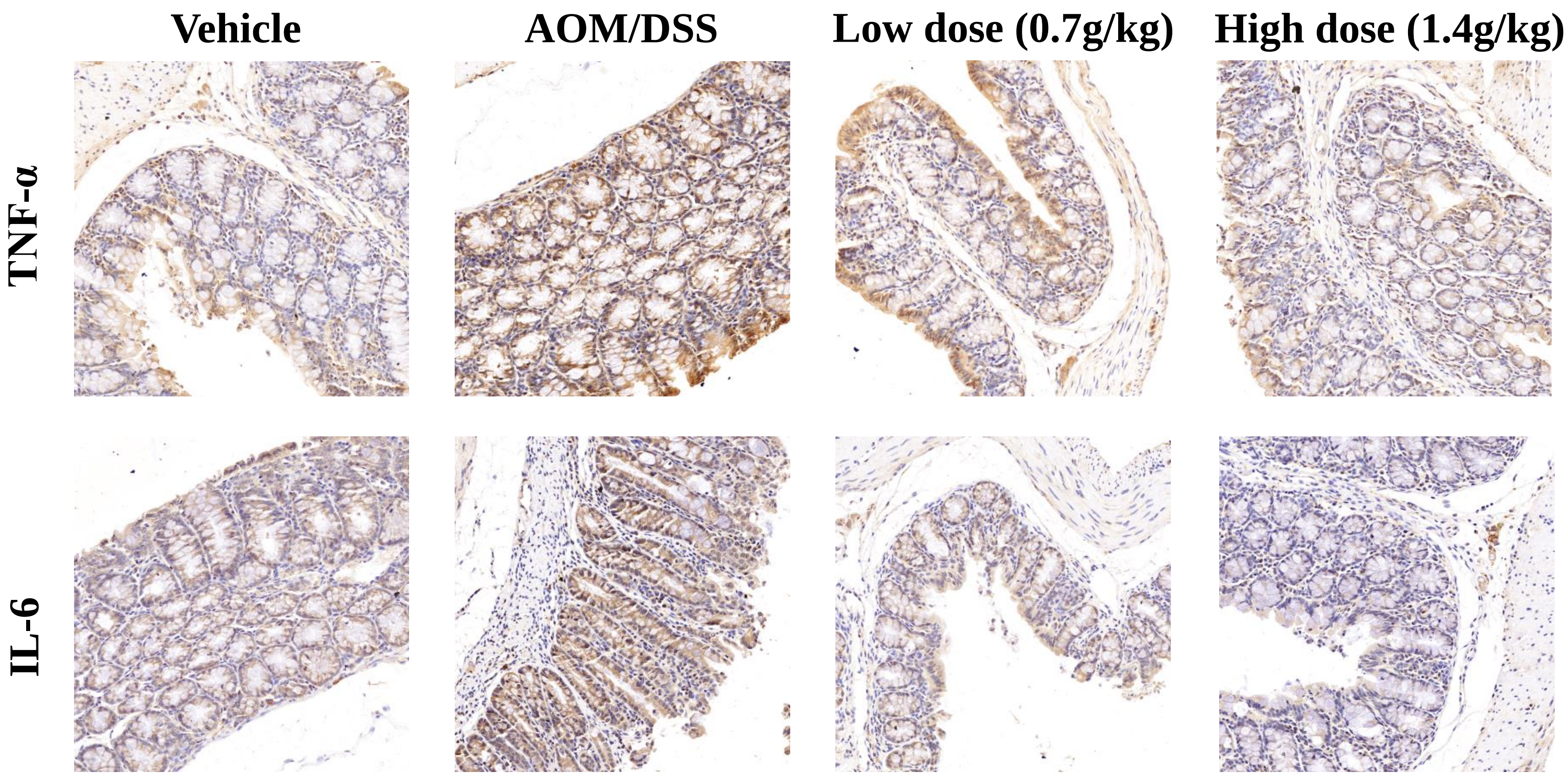

Figure S1

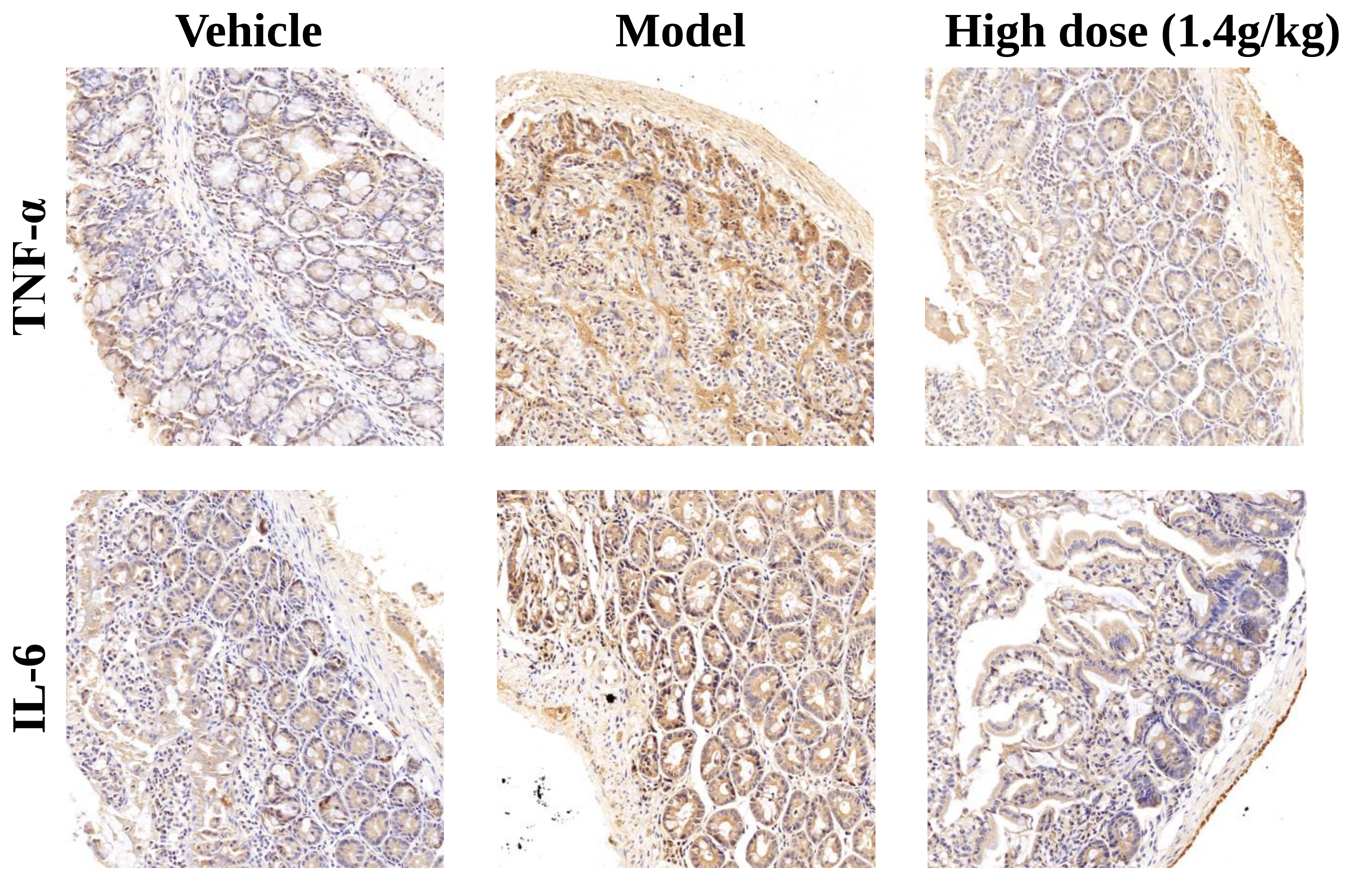

Figure S2

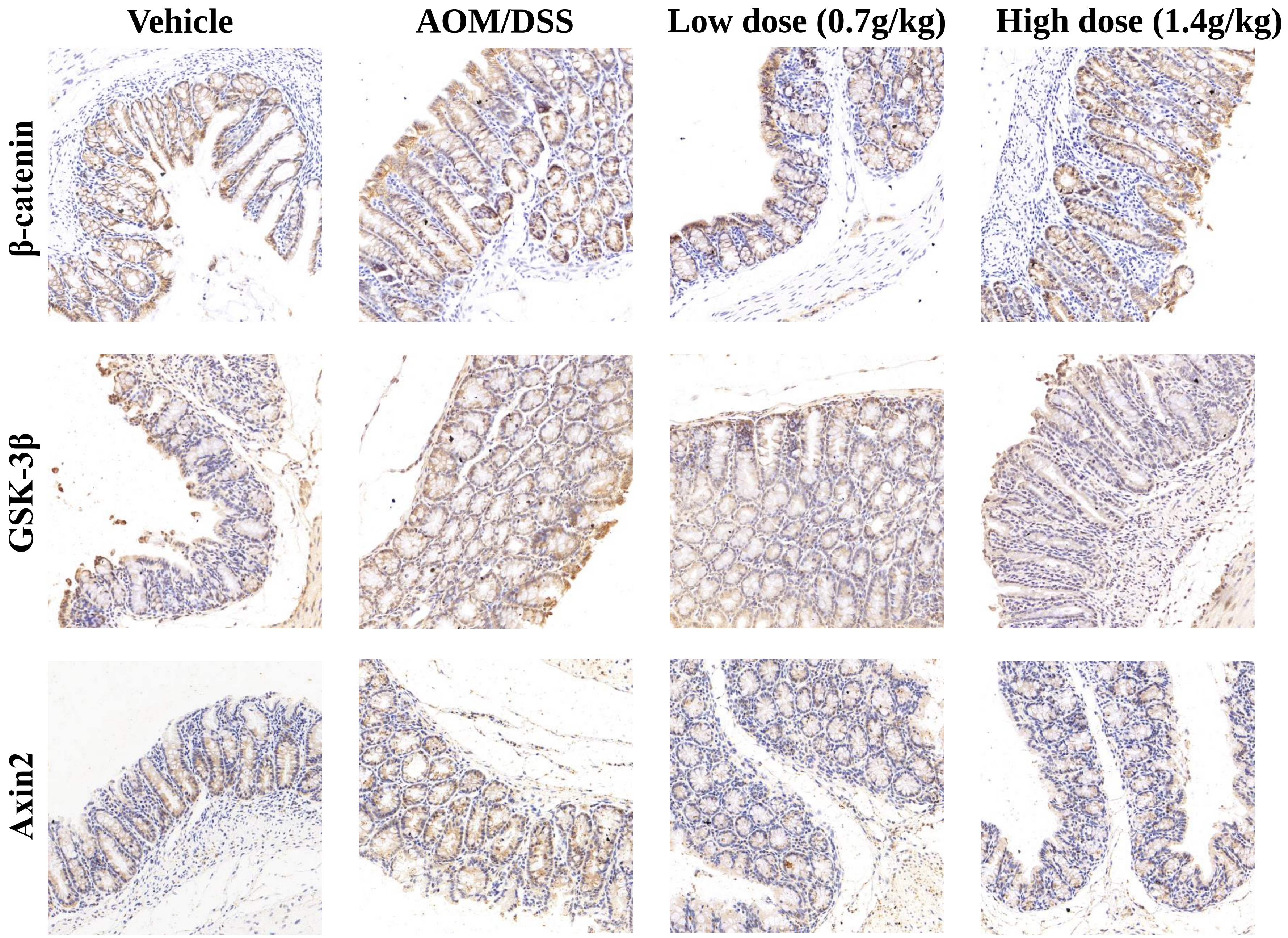
Vehicle
AOM/DSS
Low dose (0.7g/kg)
High dose (1.4g/kg)
β-catenin
GSK-3β
Axin2

Figure S2

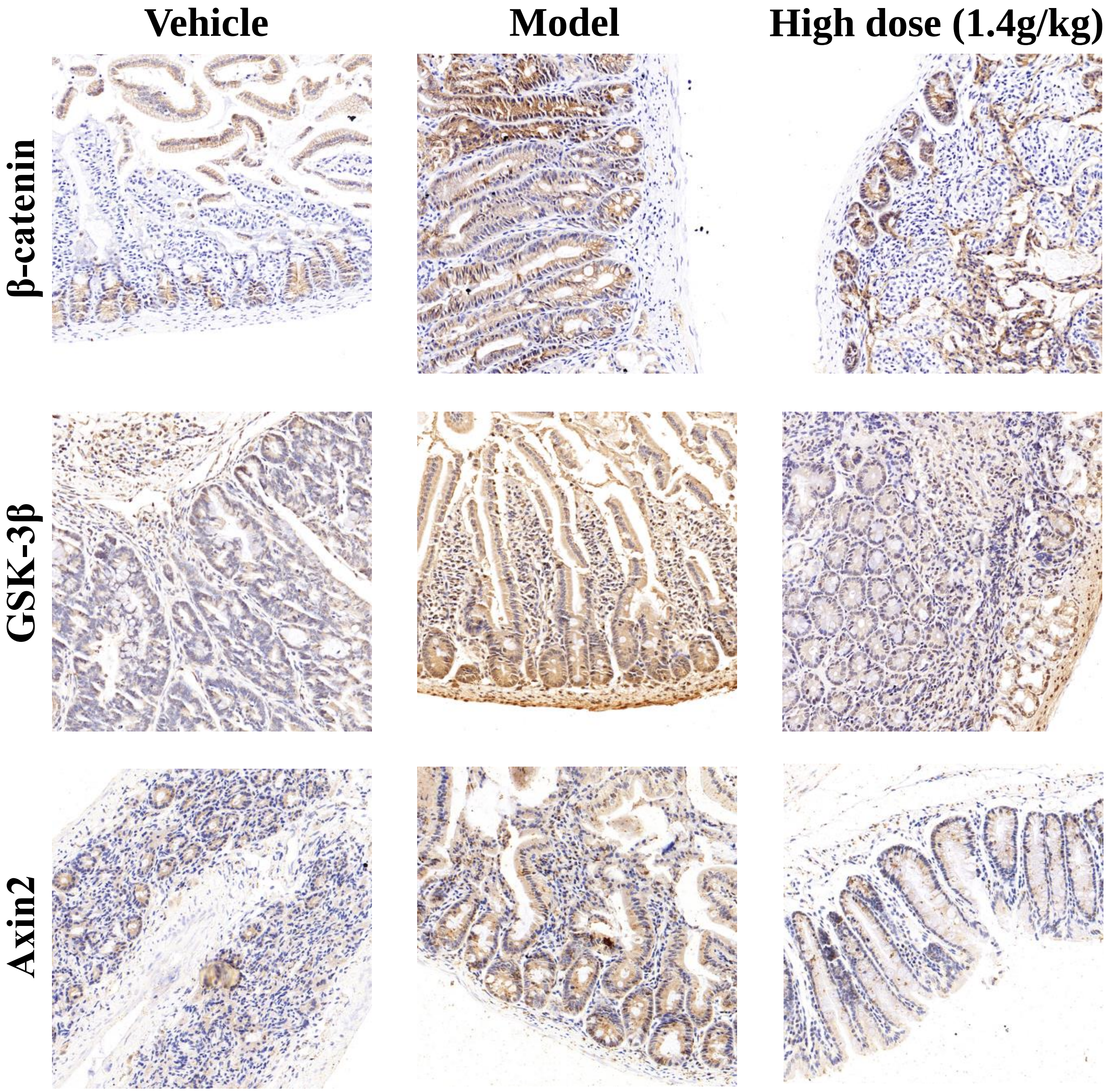

Supplement: Supplementary file 1 [file DataSheet2.PDF]
