# Supplementary material for: Qingchang Wenzhong Decoction Prevents the Occurrence of Intestinal Tumors by Regulating Intestinal Microbiota and Gasdermin E
Source: Front Physiol. 2022 Jul 14;13:917323. doi: 10.3389/fphys.2022.917323 (PMC9329543; doi:10.3389/fphys.2022.917323)
Supplement: Supplementary file 2 [file DataSheet1.PDF]

Supplementary Material

1 Supplementary Figures

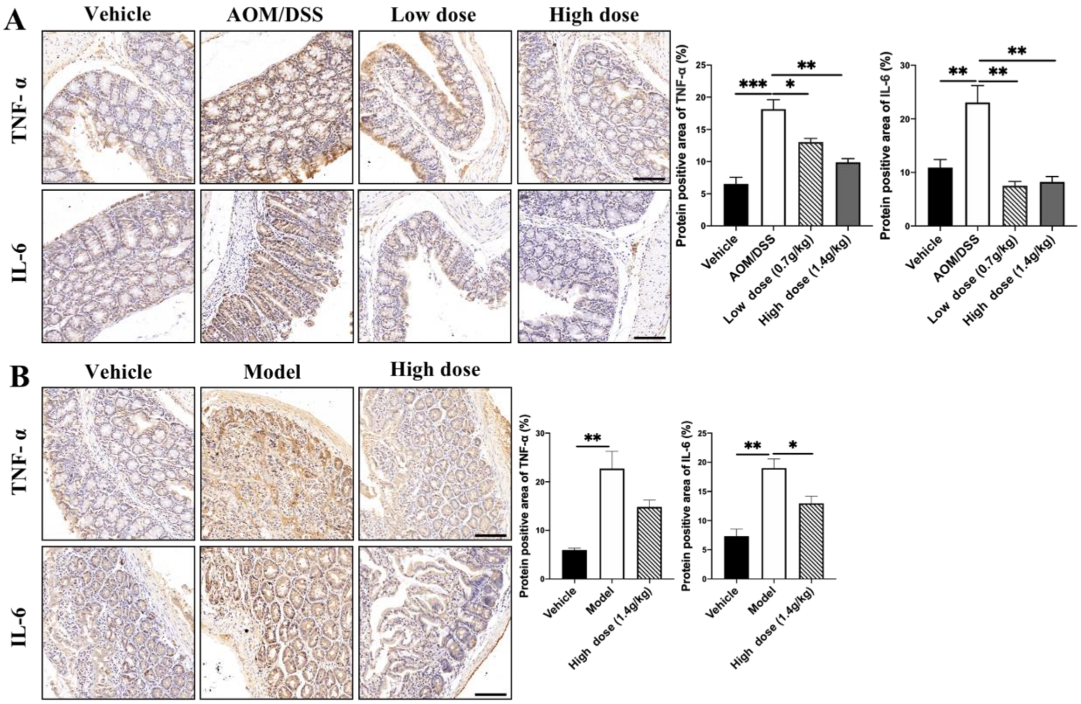

Supplementary Figure 1. Effects of QCWZD on inflammation (**A**) Levels of TNF-α and IL-6 in AOM/DSS mice (magnification: 200×) (**B**) Levels of TNF-α and IL-6 in $APC^{min/+}$ mice (magnification: 200×) High dose: 1.4 g/kg; $^{*}P<0.05$, $^{**}P<0.01$, $^{***}P<0.001$

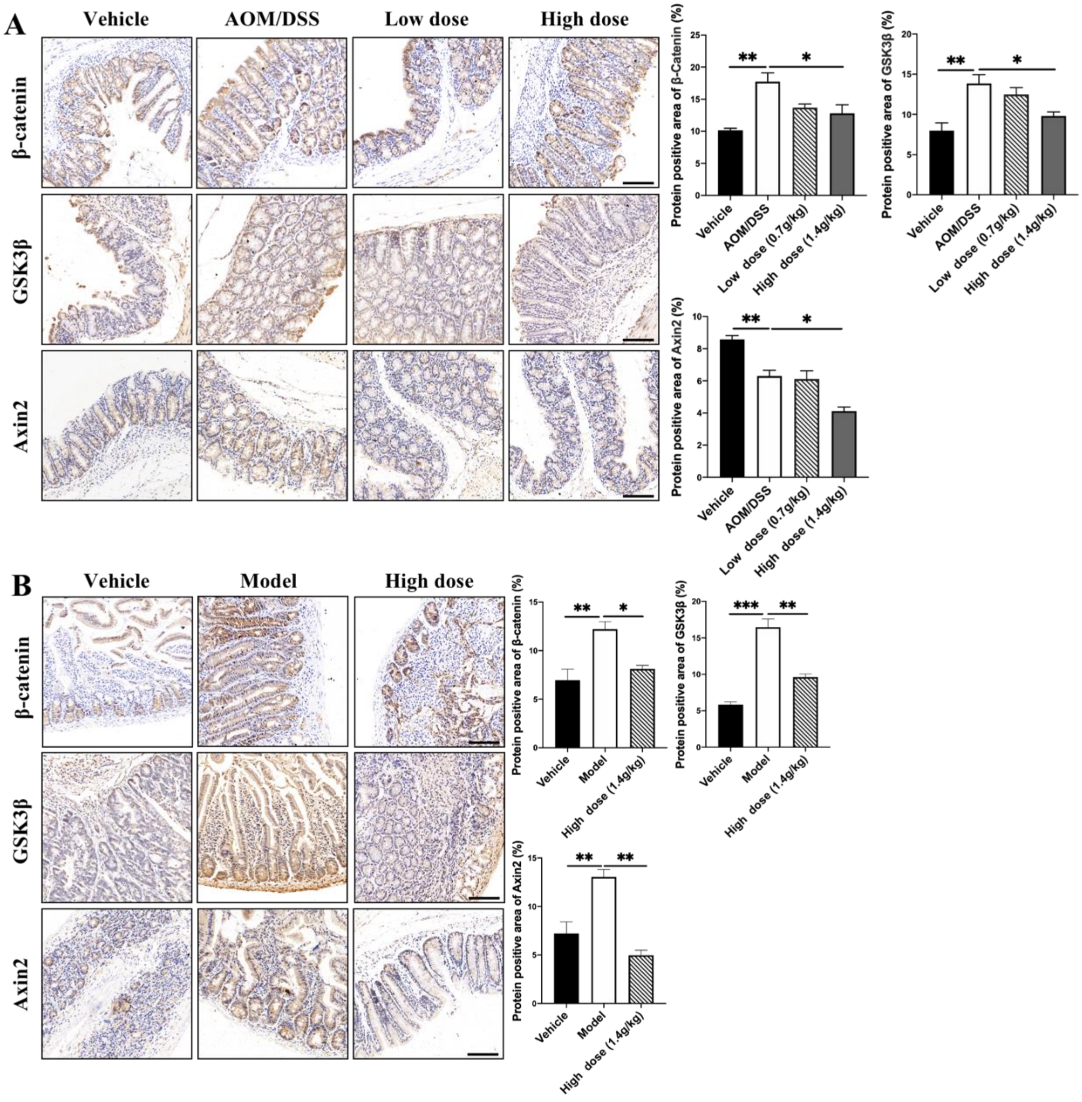

Supplementary Figure 2. Effects of QCWZD on Wnt/β-catenin signaling in the intestine (**A**) Levels of β-catenin, GSK3β, and Axin2 in AOM/DSS mice (magnification: 200×) (**B**) Levels of β-catenin, GSK3β, and Axin2 in $APC^{min/+}$ mice (magnification: 200×)
High dose: 1.4 g/kg; $^{*}P$<0.05, $^{**}P$<0.01, $^{***}P$<0.001
